# Retinal ganglion cell vulnerability to pathogenic tau in Alzheimer’s disease

**DOI:** 10.1186/s40478-025-01935-y

**Published:** 2025-02-15

**Authors:** Miyah R. Davis, Edward Robinson, Yosef Koronyo, Elena Salobrar-Garcia, Altan Rentsendorj, Bhakta P. Gaire, Nazanin Mirzaei, Rakez Kayed, Alfredo A. Sadun, Alexander V. Ljubimov, Lon S. Schneider, Debra Hawes, Keith L. Black, Dieu-Trang Fuchs, Maya Koronyo-Hamaoui

**Affiliations:** 1 Department of Neurosurgery, Maxine Dunitz Neurosurgical Research Institute, Cedars-Sinai Medical Center, 127 S. San Vicente Blvd., A6212, Los Angeles, CA 90048 USA; 2 Institute of Ophthalmologic Research Ramón Castroviejo, Complutense University of Madrid, Madrid, 28040 Spain; 3 Department of Immunology, Ophthalmology and ENT, Faculty of Optics and Optometry, Complutense University of Madrid, Madrid, 28040 Spain; 4 Health Research Institute, Clinico San Carlos Hospital (IdISSC), Madrid, 28040 Spain; 5 Mitchell Center for Neurodegenerative Diseases, University of Texas Medical Branch, Galveston, TX USA; 6 Departments of Neurology, Neuroscience, and Cell Biology, University of Texas Medical Branch, Galveston, TX USA; 7 Department of Ophthalmology, David Geffen School of Medicine, University of California Los Angeles, Los Angeles, CA USA; 8 Doheny Eye Institute, Los Angeles, CA USA; 9 Eye Program, Board of Governors Regenerative Medicine Institute, Cedars-Sinai Medical Center, Los Angeles, CA USA; 10 Department of Biomedical Sciences, Division of Applied Cell Biology and Physiology, Cedars- Sinai Medical Center, Los Angeles, CA USA; 11 Alzheimer’s Disease Research Center, Keck School of Medicine, University of Southern California, Los Angeles, CA USA; 12 Department of Neurology, Cedars-Sinai Medical Center, Los Angeles, CA USA

**Keywords:** Alzheimer’s disease, Eye, Tau protein, Amyloid beta, Ganglion cell layer, Retinal ganglion cells

## Abstract

Pathological tau isoforms, including hyperphosphorylated tau at serine 396 (pS396-tau) and tau oligomers (Oligo-tau), are elevated in the retinas of patients with mild cognitive impairment (MCI) due to Alzheimer’s disease (AD) and AD dementia. These patients exhibit significant retinal ganglion cell (RGC) loss, however the presence of tau isoforms in RGCs and their impact on RGC integrity, particularly in early AD, have not been studied. Here, we analyzed retinal superior temporal cross-sections from 25 MCI or AD patients and 16 age- and sex-matched cognitively normal controls. Using the RGC marker ribonucleic acid binding protein with multiple splicing (RBPMS) and Nissl staining, we found a 46–56% reduction in RBPMS^+^ RGCs and Nissl^+^ neurons in the ganglion cell layer (GCL) of MCI and AD retinas (*P* < 0.05–0.001). RGC loss was accompanied by soma hypertrophy (10–50% enlargement, *P* < 0.05–0.0001), nuclear displacement, apoptosis (30–50% increase, *P* < 0.05–0.01), and prominent expression of granulovacuolar degeneration (GVD) bodies and GVD-necroptotic markers. Both pS396-tau and Oligo-tau were identified in RGCs, including in hypertrophic cells. PS396-tau^+^ and Oligo-tau^+^ RGC counts were significantly increased by 2.1–3.5-fold in MCI and AD retinas versus control retinas (*P* < 0.05–0.0001). Tauopathy-laden RGCs strongly inter-correlated (*r*_*P*_=0.85, *P* < 0.0001) and retinal tauopathy associated with RGC reduction (*r*_*P*_=-0.40–(-0.64), *P* < 0.05–0.01). Their abundance correlated with brain pathology and cognitive deficits, with higher tauopathy-laden RGCs in patients with Braak stages (V–VI), clinical dementia ratings (CDR = 3), and mini-mental state examination (MMSE ≤   26) scores. PS396-tau^+^ RGCs in the central and mid-periphery showed the closest associations with disease status, while Oligo-tau^+^ RGCs in the mid-periphery exhibited the strongest correlations with brain pathology (NFTs, Braak stages, ABC scores; *r*_*S*_=0.78–0.81, *P* < 0.001–0.0001) and cognitive decline (MMSE; *r*_*S*_=-0.79, *P* = 0.0019). Overall, these findings identify a link between pathogenic tau in RGCs and RGC degeneration in AD, involving apoptotic and GVD-necroptotic cell death pathways. Future research should validate these results in larger and more diverse cohorts and develop RGC tauopathy as a potential noninvasive biomarker for early detection and monitoring of AD progression.

**Supplementary Information:**

The online version contains supplementary material available at 10.1186/s40478-025-01935-y.

## Introduction

Alzheimer’s disease (AD), the most prevalent and progressive form of senile dementia, affects an estimated 6.9 million Americans aged 65 and older [1]. It is characterized by the accumulation of amyloid beta-protein (Aβ) deposits and abnormal tau protein aggregates in the brain [18, 51]. During AD progression, microtubule-associated tau proteins undergo hyperphosphorylation (p-tau) and form toxic oligomers that spread between neurons, accelerating disease progression [14, 41, 42, 55, 59, 67]. These tau species eventually aggregate into neurofibrillary tangles (NFTs) [82], disrupting cellular functions and axonal transport, which leads to synaptic dysfunction and neuronal death [86, 99, 109, 123]. The presence of abnormal tau strongly correlates with the progression of neurodegeneration and cognitive deficits in AD [20, 37, 41, 52, 68]. AD neuropathology develops many years before neurobehavioral and cognitive disturbances become salient [52, 114, 115, 132]. Therefore, early identification of AD pathological hallmarks in the central nervous system (CNS) is crucial for early intervention and disease management.

The retina, a posterior neurosensory eye tissue, is an extension of the brain and shares many structural and functional features. Recent studies have revealed the genetic basis for eye-brain connections, suggesting bidirectional genetic causal links between retinal structures and neurological disorders, including AD [34, 97, 135]. Growing evidence indicates the presence of AD-related pathological features in the retinas of patients with mild cognitive impairment (MCI due to AD) and/or AD dementia, including various abnormal Aβ and tau species, vascular damage, micro- and macro-gliosis, and neurodegeneration [2, 5, 7, 8, 16, 17, 22, 28, 30–32, 38, 40, 43, 46, 48, 63–65, 70, 73, 74, 84, 88, 89, 98, 107, 108, 110–112, 118, 129, 131]. Regarding tauopathy, a wide range of abnormal tau isoforms have been identified in the retinas of AD patients, including pretangles and mature tangle forms: 3- and 4-repeat tau, p-tau, and citrullinated tau forms, oligomeric tau (Oligo-tau), paired helical filaments of tau (PHF-tau), as well as paperclip folding of tau and NFT-like structures [28, 30, 40, 45, 46, 63, 89, 112, 129]. We recently found that the retinas of patients with MCI (due to AD) and/or AD dementia exhibit significant increases in pathogenic p-tau at specific epitopes, including S202/T205, S214, S396, S404, and T231, as well as citrullinated R209-tau and tau oligomers (T22-positive), alongside PHF^+^ and MC-1^+^ pretangle and mature tau tangles. Epitopes S199 and T212/S214 did not show similar changes [112]. In particular, Oligo-tau and pS396-tau isoforms, commonly elevated in AD brains [100, 127], were consistently increased in MCI and AD retinas and were strongly associated with more severe brain pathology, advanced disease stages, and cognitive decline [112]. However, the impact of AD-related tauopathy on specific retinal cell types in these patients has yet to be described.

Retinal ganglion cells (RGCs) are neurons located in the retinal ganglion cell layer (GCL; as seen in optical coherence tomography – OCT imaging) and exist in various subtypes, such as midget, parasol, bistratified, and melanopsin-containing intrinsically photosensitive RGCs (mRGCs). These cells serve diverse functions, including high spatial frequency resolution, color differentiation, low spatial frequency contrast, and photoentrainment of the hypothalamus, which regulates circadian rhythms [106, 128]. Dendritic protrusions from the RGC soma receive synaptic input from the axons of bipolar and amacrine cells in the inner plexiform layer (IPL). The RGCs project their axons to form the nerve fiber layer (NFL), which converges at the optic discs and continues as the optic nerve. This pathway ultimately transmits all visual information to the brain [56]. Notably, RGCs, located on the inner retinal surface, are uniquely positioned as neurons in the CNS that can be noninvasively imaged and quantitatively assessed in vivo with high resolution using advanced adaptive optics (AO)-OCT technology, as demonstrated in recent studies [44, 79]. This advanced imaging capability enables a detailed examination of RGC pathology and may facilitate future AD diagnosis and monitoring.

In the context of AD, pioneering studies have demonstrated the loss of RGCs in patients [15, 16, 48]. Other reports have shown visual dysfunctions, such as impaired contrast sensitivity, abnormal color discrimination, and diminished visual fields, which can be attributed to RGC degeneration [38, 54, 101, 104, 125]. Subsequent investigations into the AD retina have found thinning of the NFL, reduced density of melanopsin-containing RGCs, GCL cell loss, and elevated apoptotic markers, along with intraneuronal Aβ oligomers and other Aβ species within RGCs in these patients [5–7, 23, 26, 38, 57, 63, 64, 69, 70, 73, 77]. A recent report in several transgenic murine models of AD showed RGC susceptibility, manifested as a reduction in RGC dendritic fields, occurring in parallel with hippocampal dendritic spine loss [13]. An additional study detected an increased total tau burden in RGCs in an AD-murine model [24]. However, the vulnerability of RGCs to pathogenic tau accumulation in AD patients, particularly during the earliest stages of functional impairment (MCI due to AD), and its potential relationship with disease status, has not yet been explored.

In the current study, we addressed these gaps by investigating the density, size, and distribution of RGCs in the superior temporal retinal tissues from patients with MCI (due to AD) and AD dementia, compared with cognitively normal (CN) individuals. We examined whether RGCs in AD express markers of apoptosis, granulovacuolar degeneration (GVD) bodies, and GVD-associated necroptosis. In addition, we explored whether AD-related pathogenic tau forms, pS396-tau and Oligo-tau, are specifically present within RGCs, and quantified RGCs containing these abnormal tau isoforms in this cohort. The interplay between RGC integrity and pS396-tau- and Oligo-tau-containing RGCs, as well as the overall burden of Aβ and tau pathology in the retina, was assessed, and correlations with disease status were determined. Our analyses indicated an early and substantial decline in RGCs, which was associated with increased pS396-tau- and Oligo-tau-laden RGCs in MCI and AD patients compared to age- and sex-matched CN controls. RGCs in AD patients exhibited hypertrophic soma and nucleus displacement, concomitant with expression of apoptotic and GVD/necroptotic markers. The levels of pS396-tau- and Oligo-tau-laden RGC counts strongly correlated with corresponding brain pathology and cognitive status.

## Materials and methods

### Postmortem eyes

Human eye and brain tissues were collected from donor patients with premortem clinical diagnoses of MCI and AD dementia (confirmed by postmortem AD neuropathology), along with age- and sex-matched CN controls (total *n* = 41 subjects). These tissues were primarily obtained from the Alzheimer’s Disease Research Center (ADRC) Neuropathology Core in the Department of Pathology (IRB protocol HS-042071) at the Keck School of Medicine, University of Southern California (USC, Los Angeles, CA). Additional eyes were obtained from the National Disease Research Interchange (NDRI, Philadelphia, PA) under the approved Cedars-Sinai Medical Center IRB protocol Pro00019393. Both USC-ADRC and NDRI maintain human tissue collection protocols approved by their managerial committees and subject to oversight by the National Institutes of Health. Histological studies at Cedars-Sinai Medical Center were performed under IRB protocols Pro00053412 and Pro00019393. Demographic, clinical, and neuropathological information on human donors is detailed in Tables 1 and Suppl. Table 1. Subjects with macular degeneration, glaucoma, and diabetic retinopathy were excluded from this study. The available retinal tissues from individual donors are specified in Suppl. Table 1. For the histopathological analysis, the human cohort consisted of AD dementia (*n* = 15), MCI due to AD (*n* = 10), and CN controls (*n* = 16). All patients’ identities were protected by de-identifying tissue samples, ensuring they could not be traced back to the donors.

**Table 1:** Demographic and neuropathological data on human brain and retinal donors in this study

		CN	MCI	AD	F	*P*
Retinal samples (*N* = 41)		16 10 F (63%) 6 M	10 7 F (70%) 3 M	15 8 F (53%) 7 M	-	-
Age at death (years)		80.5 ± 11.1	88.4 ± 6.6	87.5 ± 8.0	2.93	0.07
Race		14 W, 1 H, 1B	8 W, 1B, 1 H	12 W, 2 H, 1 A	-	-
PMI (h)		7.8 ± 4.5	10.1 ± 5.4	8.8 ± 4.5	0.5	0.73
MMSE score (*N* = 30)		28.7 ± 2.1	20.1 ± 7.0	13.8 ± 7.3	17.01	**< 0.0001**
CDR score (*N* = 31)		0.57 ± 0.8	2.1 ± 1.1	2.5 ± 0.9	10.53	**0.0004**
Brain neuropathology (*N* = 32)	Braak stage (%)	0-II (57%) III-IV (43%) V-VI (0%)	0-II (30%) III-IV (30%) V-VI (40%)	0-II (0%) III-IV (13%) V-VI (87%)	15.2	**< 0.0001**
	ABC average	1.37 ± 0.91	2.20 ± 0.59	2.82 ± 0.21	17.13	**< 0.0001**
	Aβ plaque (severity score)	1.12 ± 1.31	1.88 ± 0.79	2.65 ± 0.77	6.98	**0.0034**
	NFTs (severity score)	0.43 ± 0.53	1.71 ± 0.91	2.36 ± 0.70	16.06	**< 0.0001**
	NTs (severity score)	0.49 ± 0.99	1.24 ± 0.82	1.69 ± 0.90	4.27	**0.024**

List of human donors included in this study (*N* = 41 subjects). Paired brains with neuropathological assessments were available for 32 human donors. ABC scores comprise of mean grades for: (A) Aβ plaque score modified from Thal, (B) NFT stage modified from Braak, and (C) neuritic plaque score modified from CERAD. Group values are presented as mean ± standard deviation. *F* and P-values were determined using one-way analysis of variance (ANOVA) with Tukey’s multiple comparisons test. P-values presented in bold type demonstrate statistical significance. Abbreviations: Aβ, amyloid beta-protein; AD, Alzheimer’s disease; A, Asian; B, Black; CDR, Clinical Dementia Rating; CN, cognitively normal controls; F, female; H, Hispanic; M, male; MCI, mild cognitive impairment; MMSE, Mini-Mental State Examination; NFTs, neurofibrillary tangles; NTs, neuropil threads; PMI, postmortem interval; W, White

### Clinical and neuropathological assessments

The ADRC provided clinical and neuropathological reports on patients’ neurological examinations, neuropsychological and cognitive tests, family history, and medication lists, as collected in the ADRC system using the Uniform Data Set (UDS) [12]. The NDRI provided the medical history of additional patients. Most cognitive evaluations were performed annually and, in most cases, less than one year prior to death. Cognitive testing scores from evaluations made closest to the patient’s death were used for this analysis. Two global indicators of cognitive status were used for clinical assessment: the Clinical Dementia Rating (CDR scores: 0 = normal; 0.5 = very mild impairment; 1 = mild dementia; 2 = moderate dementia; or 3 = severe dementia) [85] and the Mini-Mental State Examination (MMSE scores: 24–30 = CN; 20–23 = MCI; 10–19 = moderate dementia; or 9 ≥ severe dementia) [35]. In this study, the composition of the clinical diagnostic groups (AD, MCI, or CN) was determined by source clinicians based on a comprehensive battery of tests, including neurological examinations, neuropsychological evaluations, and the cognitive tests. Specifically, the diagnosis of MCI due to AD was assigned to patients who had an antemortem clinical diagnosis of MCI (based on the comprehensive battery of behavioral and cognitive tests) caused by AD. These patients had a postmortem confirmation of AD neuropathology (according to the ADNC—Alzheimer’s disease neuropathological change guidelines) and showed no evidence of other diseases, such as Lewy body dementia, Parkinson’s disease, FTD/FTLD (PSP or Pick’s disease), or cognitive impairment due to stroke or small vessel disease.

To obtain a final diagnosis based on the neuropathological reports, we used the modified Consortium to Establish a Registry for Alzheimer’s Disease (CERAD) criteria [80, 103], as outlined in the National Institute on Aging (NIA)/Regan protocols with revisions by the NIA and Alzheimer’s Association [50]. The assessment included Aβ burden (measured as diffuse, immature, or mature plaques), amyloid angiopathy, neuritic plaques, NFTs, neuropil threads (NTs), granulovacuolar degeneration, Lewy bodies, Hirano bodies, Pick bodies, balloon cells, neuronal loss, microvascular changes, and gliosis. These pathologies were assessed in multiple brain areas, including the hippocampus (particularly the Cornu ammonis CA1, at the level of the thalamic lateral geniculate body), entorhinal cortex, superior frontal gyrus of the frontal lobe, superior temporal gyrus of the temporal lobe, superior parietal lobule of the parietal lobe, primary visual cortex (Brodmann Area-17), and visual association (Area-18) of the occipital lobe. In all cases, uniform brain sampling was conducted by a neuropathologist.

Cerebral amyloid plaques, NFTs, and NTs were evaluated using anti–β-amyloid mAb clone 4G8 immunostaining, Thioflavin-S (ThioS) histochemical staining, and Gallyas silver staining in formalin-fixed, paraffin-embedded tissue sections. The ADRC neuropathologists assigned severity scores based on semi-quantitative observations. The scale for Aβ/neuritic plaques was determined by the presence of 4G8- and/or Thioflavin-S-positive and/or Gallyas silver-positive plaques measured per 1 mm^2^ of brain area (0 = none; 1 = sparse [≤ 5 plaques]; 3 = moderate [6–20 plaques]; 5 = abundant/frequent [21–30 plaques or greater]; or N/A = not applicable), as previously described [83] in the NACC NP Guidebook, Version 10, January 2014: https://naccdata.org/data-collection/forms-documentation/np-10. The brain NFT or NT severity scoring system was derived from observed burden of these AD neuropathologic changes, as detected by Gallyas silver and/or Thioflavin-S staining [82, 83, 126], and measured per 1 mm^2^ of brain area. The assigned NFT or NT scores were as follows: 0 = none; 1 = sparse (mild burden); 3 = moderate (intermediate burden); or 5 = frequent (severe burden). For both histochemical and immunohistochemical staining, each anatomical area of interest was assessed for relevant pathology using a 20X objective (200X high power magnification), and representative fields were graded using the semiquantitative scale as detailed above. Validation of AD neuropathic change (ADNC), especially NTs, was performed using a 40X objective (400X high power magnification), and an average of two readings was assigned to each individual patient.

A final diagnosis of AD neuropathological change was determined using an “ABC” score derived from three separate 4-point scales. We used the modified Aβ plaque Thal score (A0 = no Aβ or amyloid plaques; A1 = Thal phase 1 or 2; A2 = Thal phase 3; or A3 = Thal phase 4 or 5) [122]. For the NFT stage, we applied the modified Braak staging for silver-based histochemistry or p-tau IHC (B0 = no NFTs; B1 = Braak stage I or II; B2 = Braak stage III or IV; or B3 = Braak stage V or VI) [19]. For neuritic plaques, we used the modified CERAD score (C0 = no neuritic plaques; C1 = CERAD score sparse; C2 = CERAD score moderate; or C3 = CERAD score frequent) [80]. Neuronal loss, gliosis, granulovacuolar degeneration, Hirano bodies, Lewy bodies, Pick bodies, and balloon cells were all evaluated (0 = absent; 1 = present) in multiple brain areas by staining tissues with hematoxylin and eosin (H&E). Brain atrophy was evaluated (0 = none; 1 = mild; 3 = moderate; 5 = severe; or 9 = not applicable).

### Processing of eye globes and retinal tissues

The processing of eye globes, isolation and preparation of retinal strips, and retinal immunostaining were extensively detailed in [63, 64, 112]. Briefly, donor eyes were collected within an average of 9 h after death, puncture at the limbus and fixed in 10% neutral buffered formalin (NBF) or 4% paraformaldehyde (PFA) then stored at 4 °C. Regardless of the source of the human donor eye (USC-ADRC or NDRI), the same tissue collection and processing methods were applied.

### Preparation of retinal strips

Eyes fixed in 10% NBF or 4% PFA were dissected as previously described [63, 64, 112]. Flatmounts were prepared after careful dissection of the eye globes and thorough cleaning of the vitreous humor. Flatmount strips (~ 2 mm wide) extending diagonally from the optic disc (OD) to the ora serrata (~ 20–25 mm long) were prepared in 4 predefined regions: Superior Temporal (ST), Inferior Temporal (IT), Inferior Nasal (IN), and Superior Nasal (SN). In this study, we focused our analysis on the ST retinal strip due to the high presence of AD pathology in this region [63, 64, 112]. The flatmount-derived strips were then paraffinized using standard techniques and embedded in paraffin after flip-rotating 90° horizontally. The retinal strips were sectioned (7–10 μm thick) and mounted on microscope slides coated with (3-Aminopropyl) triethoxysilane. This sample preparation technique allowed for extensive and consistent access to retinal quadrants, layers, and pathological subregions.

### Immunofluorescent staining

Retinal sections were deparaffinized using 100% xylene twice (10 min each), rehydrated with decreasing concentrations of ethanol (100–70%), and washed with distilled water followed by PBS. After deparaffinization, tissue sections were treated with target retrieval solution (pH 6.1; S1699, Dako) at 98 °C for 1 h and then washed with PBS. Next, tissues were incubated in blocking buffer (Dako #X0909) supplemented with 0.1% Triton X-100 (Sigma, T8787) for 1 h at room temperature (RT), followed by overnight incubation with primary antibody (Ab) at 4 °C (Abs information provided in Suppl. Table 2). The sections were then washed three times with PBS and incubated with secondary Abs against each species (1:200, Suppl. Table 2) for 1 h at RT. After rinsing with PBS three times, the sections were mounted with ProLong Gold antifade reagent with DAPI (Thermo Fisher #P36935).

### Peroxidase-based immunostaining

After deparaffinization and antigen retrieval treatment, the tissues were treated with 70% formic acid (ACROS) for 10 min at room temperature. The tissues were then washed with wash buffer (Dako S3006) supplemented with 0.1% Triton X-100 (Sigma, T8787) for 1 h, followed by treatment with H_2_O_2_ for 10 min and a rinse with wash buffer. Primary Ab (Suppl. Table 2) were diluted with background reducing components (Dako S3022) and incubated with the tissues overnight at 4 °C. The tissues were rinsed thrice with wash buffer on a shaker and incubated for 30 min at 37 °C with secondary Ab (goat anti-rabbit HRP conjugated, Dako Envision K4003), followed by three more rinses with wash buffer on a shaker. Diaminobenzidine (DAB) substrate (Dako K3468) was then applied. Some slides were counterstained with hematoxylin and mounted with Faramount aqueous mounting medium (Dako, S3025). Routine controls were processed using an identical protocol, while omitting the primary antibodies to assess nonspecific labeling.

### Nissl staining

A basic (alkaline) dye was used to label nuclei and granules (i.e., ribosomal RNA) in neurons. The cytoplasm of neurons is specifically stained with the Nissl staining technique, while the perikarya of other cellular elements are either weakly visualized or not at all [53]. Deparaffinized and rehydrated sections were stained in 0.1% Cresyl Violet acetate (Sigma #C5042) for 5 min, rapidly rinsed in tap water, and briefly dipped in 70% ethanol. The sections were then dehydrated through 2 changes of absolute ethanol for 3 min each, followed by immersion in xylene twice for 2 min and mounted in mounting medium xylene (Fisher scientific company, L.L.C. #245–691). An average of 12 images (from the superior quadrant), covering the retinal neurons from the optic disc to the ora serrata, were captured at a 20x objective and analyzed to quantify the area and number of retinal GCL neurons.

### Microscopy and stereological quantification

Fluorescence and brightfield images were acquired using a Carl Zeiss Axio Imager Z1 fluorescence microscope (with motorized Z-drive) equipped with ApoTome, AxioCam HRc, and AxioCam MRm monochrome cameras (version 3.0; resolution of 1388 × 1040 pixels, 6.45 μm × 6.45 μm pixel size, and a dynamic range of > 1:2200, which delivers low-noise images due to a Peltier-cooled sensor) with ZEN 2.6 blue edition software (Carl Zeiss MicroImaging, Inc.). Multi-channel image acquisition was used to create images with multiple channels. Images were consistently captured at the same focal planes with identical exposure time, using a 20x objective at a resolution of 0.25 μm. Approximately 15 images were obtained from each retina. The acquired images were converted to grayscale and standardized to baseline using a histogram-based threshold in Fiji ImageJ (NIH) software (version 1.53c). For each biomarker, the total area of immunoreactivity was determined using the same threshold percentage from the baseline in ImageJ (with the same percentage threshold setting for all diagnostic groups). The images were then subjected to particle analysis to determine the immunoreactive (IR) area and/or area fraction (%). For each immunoreactivity quantification, all slides and microscopic images were processed and acquired in a single batch.

### RGC soma size measurement

The size of RGC somas was measured using Fiji ImageJ (NIH) software (version 1.53c) with the polygonal selection tool. For each 20x retinal image (10–12 images per patient), the soma area of the top 3 largest RBPMS^+^ RGCs was measured; 30–36 RGCs per patient were analyzed. In addition, all identifiable RBPMS^+^ RGCs were manually assessed in each field throughout the entire retinal strips (a total of 2,051 cells). RGC soma area measurements of all cells and the average RGC soma area per patient, across all retinal subregions, were plotted for each diagnostic group and subjected to statistical analysis.

### Statistical analysis

GraphPad Prism Software version 9.5.1 was used for statistical analyses. One-way or two-way ANOVA followed by Tukey’s multiple comparison post-test was used to determine statistical significance between three or more groups. Two-group comparisons were analyzed using a two-tailed unpaired Student’s *t-test*. The statistical association between two variables was determined by Pearson’s correlation (parametric) or Spearman’s rank correlation (non-parametric) analyses. Pair-wise Pearson’s (*r*_P_) or Spearman’s (*r*_S_) coefficients, with unadjusted P values, were used to indicate the direction and strength of the linear relationship between the two variables. Results are expressed as the mean ± standard deviation (SD) in tables, as median, lower, and upper quartiles in violin plots and mean ± standard error of the mean (SEM) in bar graphs. Degrees of significance are presented as: *P < 0.05, **P < 0.01, ***P < 0.001, and ****P < 0.0001. Data analysis was conducted using coded identifiers, and analysts remained blinded to the diagnostic groups until all analyses were completed.

## Results

**Fig. 1:**
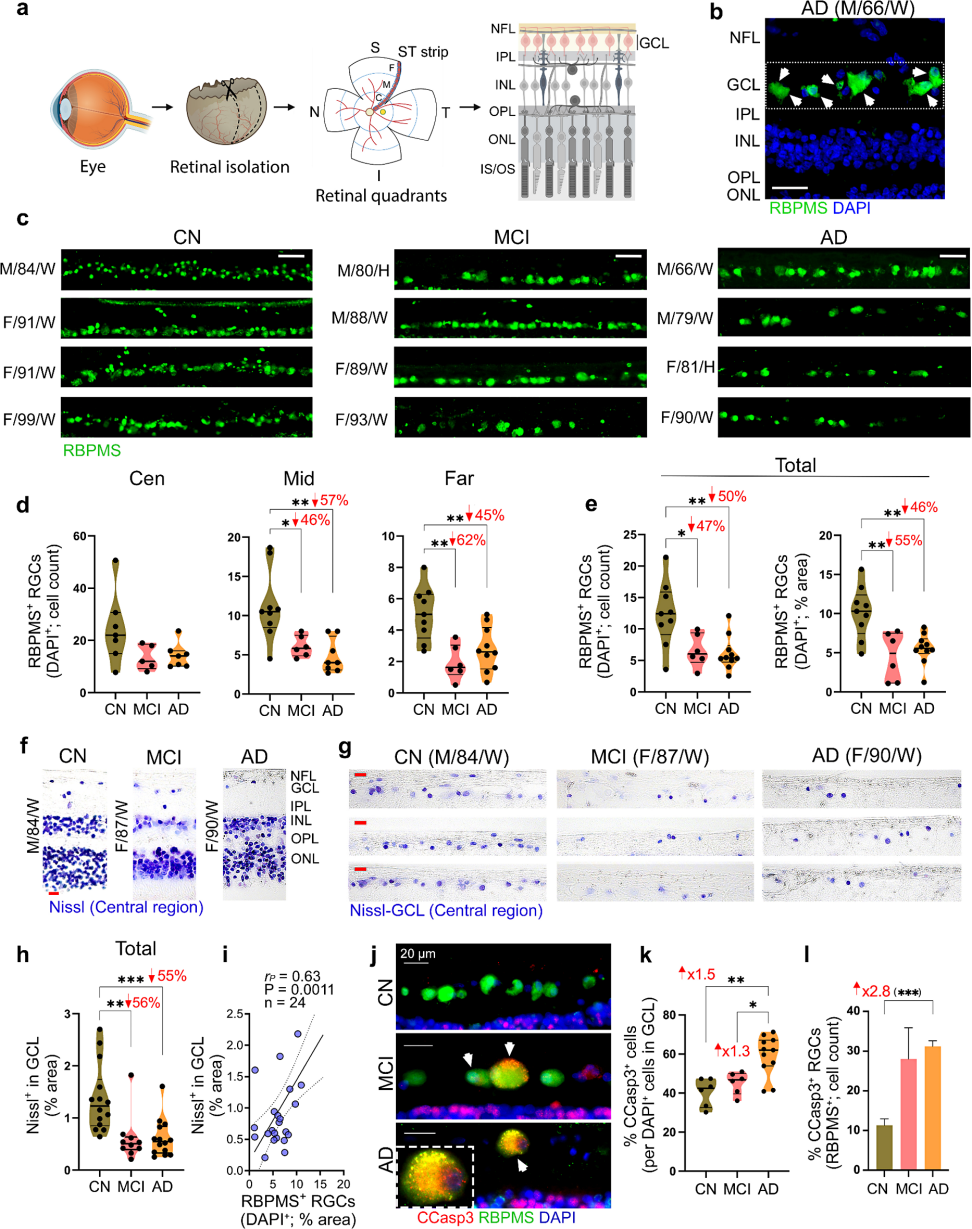
Ganglion cell integrity in retinal tissues of MCI and AD patients. (**a**) Illustration of the histological process, including retinal isolation, cross-section preparation, and analysis of the superior temporal (ST) strip, extending from the optic disc to the ora serrata and anatomically predefined into central (Cen, C), middle (Mid, M) and far-peripheral (Far, F) subregions. The retinal ganglion cell layer (GCL) was analyzed in this study. (**b**) Microscopic image of a retinal cross-section from an AD patient, immunolabeled with retinal ganglion cell (RGC)-specific marker, ribonucleic acid binding protein with multiple splicing (RBPMS; green), and nuclei labelling with DAPI (blue). Scale bar: 25 μm. (**c**) Representative microscopic images of RGCs within the GCL, labeled with RBPMS (green), in retinal cross-sections from patients with mild cognitive impairment (MCI due to AD, *n* = 4) and Alzheimer’s disease (AD) dementia (*n* = 4), and cognitively normal individuals (CN, *n* = 4). Scale bar: 50 μm. (**d**, **e**) Violin plots display quantitative immunohistochemistry analyses of RBPMS^+^DAPI^+^ RGCs by (**d**) cell count in Cen, Mid- and Far-peripheral subregions, and (**e)** cell count (left) and percent area (right) in the total ST region (*n* = 25 subjects; *n* = 9 CN, *n* = 6 MCI, *n* = 10 AD). (**f**, **g**) Representative microscopic images of retinal cross-sections from CN, MCI, and AD donors labeled with Nissl (purple) in (**f)** all analyzed retinal layers (ONL to NFL) and (**g**) GCL separately, in the Central ST subregion. Scale bars: 20 μm. (**h**) Quantitative analyses of Nissl^+^ percent area in the GCL for the total ST region (*n* = 38 subjects; *n* = 14 CN, *n* = 10 MCI, *n* = 14 AD). (**i**) Pearson’s correlation coefficient (*r*_*P*_) analysis between RBPMS^+^ RGCs percent area and Nissl^+^ cells (in GCL) percent area. (**j**) Representative microscopic images of the early apoptotic cell marker, cleaved caspase-3 (CCasp3^+^, red) in RBPMS^+^ cells (green) with nuclei DAPI (blue) in the GCL of CN, MCI and AD donors. (**k**) Quantitative analysis of the percent area of CCasp3^+^ in the GCL, normalized to nuclei count (*n* = 23 subjects; *n* = 6 CN, *n* = 6 MCI, *n* = 11 AD). (**l**) Percentage of CCasp3^+^RBPMS^+^ RGC count in a subset of the same cohort. The bar graph displays mean ± SEM. Violin plots show individual data points and median, lower, and upper quartiles. *P < 0.05, **P < 0.01, ***P < 0.001, ****P < 0.0001, by one-way or two-way ANOVA followed by Tukey’s post-hoc multiple comparison test or unpaired Student t-test (in parenthesis). Percent decreases and fold changes are shown in red. F, female; M, male; Age (in years); Ethnicity: W, White and H, Hispanic; NFL, Nerve fiber layer; IPL, Inner Plexiform Layer; INL, Inner Nuclear Layer; OPL, Outer Plexiform Layer; ONL, Outer Nuclear Layer; IS/OS, inner segment and outer segment. Illustrations created with Biorender.com

We investigated the integrity of RGCs, including their number, morphology, and distribution, in relation to abnormal retinal tau isoforms and their accumulation within RGCs in early and advanced-stage AD. Retinal superior temporal (ST) cross-sections (Fig. 1a, b) were analyzed from MCI due to AD (*n* = 10, mean age 88.4 ± 6.6 years, 7 females/3 males), AD dementia (*n* = 15, mean age 87.5 ± 8.0 years, 8 females/7 males), and CN controls (*n* = 16, mean age 80.5 ± 11.1 years, 10 females/6 males). Demographic, clinical, and neuropathological information is detailed in Table 1 (list of individual donor eyes and respective brains detailed in Suppl. Table 1).

### RGC loss and apoptosis in MCI and AD patients

We first assessed RGC numbers and distribution across ST subregions in a sub-cohort of patients with MCI (*n* = 6, mean age 89.5 ± 5.24 years, 3 females/3 males), AD (*n* = 10, mean age 86.0 ± 8.89 years, 4 females/6 males), and age- and sex-matched CN controls (*n* = 9, mean age 85.89 ± 11.85 years, 5 females/4 males), using a selective pan-RGC marker, ribonucleic acid binding protein with multiple splicing (RBPMS), for immunohistochemical (IHC) analysis. Previous studies have shown that, RBPMS is specifically expressed in the entire RGC population, despite the heterogeneity of other neurons under pathological conditions, including displaced amacrine cells within the GCL [91, 94, 102]. Compared to the retinas of CN individuals, the density of RGCs appeared lower in MCI and AD dementia patients, with their cytoplasm enlarged or swollen (Fig. 1c; extended data for RBPMS with DAPI nuclei staining in Suppl. Figure 1a). Quantitative analysis of RBPMS^+^ RGC counts per retinal subregion (central, mid-, and far-periphery) and the total ST region revealed substantial reductions in RGC count and percent area–ranging from 42–65%–in MCI and AD patients compared to CN individuals (Fig. 1d, e and Suppl. Figure 1b; *P* < 0.05 − 0.01). RBPMS^+^ RGC count loss in MCI and AD retinas was more significant in the mid- and far-periphery regions, which are distal from the optic nerve head.

We next examined neurodegeneration in the GCL using Nissl staining, an alkaline dye that labels nuclei and granules (i.e., ribosomal RNA) in neurons, in a sub-cohort of patients diagnosed with MCI (*n* = 10, mean age 88.4 ± 6.6 years, 7 females/3 males), AD (*n* = 15, mean age 87.5 ± 8.0 years, 8 females/7 males), and CN controls (*n* = 14, mean age 80.6 ± 12.1 years, 9 females/5 males) (Fig. 1f-i). Representative images from the central ST region showed a reduction in cell numbers across all retinal layers (Fig. 1f), particularly in the GCL (Fig. 1g), in MCI and AD patients compared to CN controls. Quantitative analysis of Nissl^+^ percent area in the GCL across retinal subregions indicated a marked 53-64% neuronal loss in the central and mid-peripheral subregions of MCI and AD patients compared to CN controls (Suppl. Figure 1c). No statistically significant reduction was observed in the far-peripheral subregion. In the total ST region, a substantial 55-56% reduction in Nissl^+^ percent area in the GCL was observed for both AD and MCI groups compared to CN controls (Fig. 1h; *P* < 0.01 − 0.001). Pearson’s correlation coefficient (*r*) analysis demonstrated a strong correlation between the two RGC integrity parameters, RBPMS^+^ RGCs and Nissl^+^ GCL residing neurons percent areas (*r* = 0.63, *P* = 0.0011; Fig. 1i). To assess whether RGCs or GCL residing neurons were lost due to apoptotic cell death mechanisms, we performed IHC using an antibody against an early apoptotic marker [124], cleaved caspase 3 (CCasp3; Fig. 1j-l). Increased CCasp3 labeling was observed in RBPMS^+^ RGCs of MCI and AD patients (Fig. 1j; extended data in Suppl. Figure 1d). Quantitative analysis of percent CCasp3^+^ GCL residing cells revealed a significant 1.5-fold and 1.3-fold increase in AD retinas compared to CN and MCI retinas, respectively (Fig. 1k; *P* < 0.05 − 0.01), with no differences noted between MCI and CN retinas. Similarly, a quantitative analysis of colocalized CCasp3^+^RBPMS^+^ RGC count showed a significant 1.8-fold increase in AD compared to MCI and CN groups (Suppl. Figure 1e; *P* < 0.05). Notably, the percent of CCasp3^+^RBPMS^+^ RGCs of the total RBPMS^+^ cell population was markedly increased by 2.8-fold in AD compared to CN, with a non-significant trend of increase in the MCI group (Fig. 1l; *P* < 0.001). This analysis showed that compared to ~ 10% RGC-expressing CCasp3 in CN individuals, this number was elevated to approximately 25–30% apoptotic RGCs in MCI and AD patients (Fig. 1l; Extended data on retinal CCasp3 immunoreactive area normalized to retinal thickness showed significant 1.5- and 3.1-fold increases in MCI and AD, respectively, as compared to CN (Suppl. Figure 1f; *P* < 0.05 and < 0.0001, respectively).

### RGC hypertrophy and GVD-associated necroptosis

**Fig. 2:**
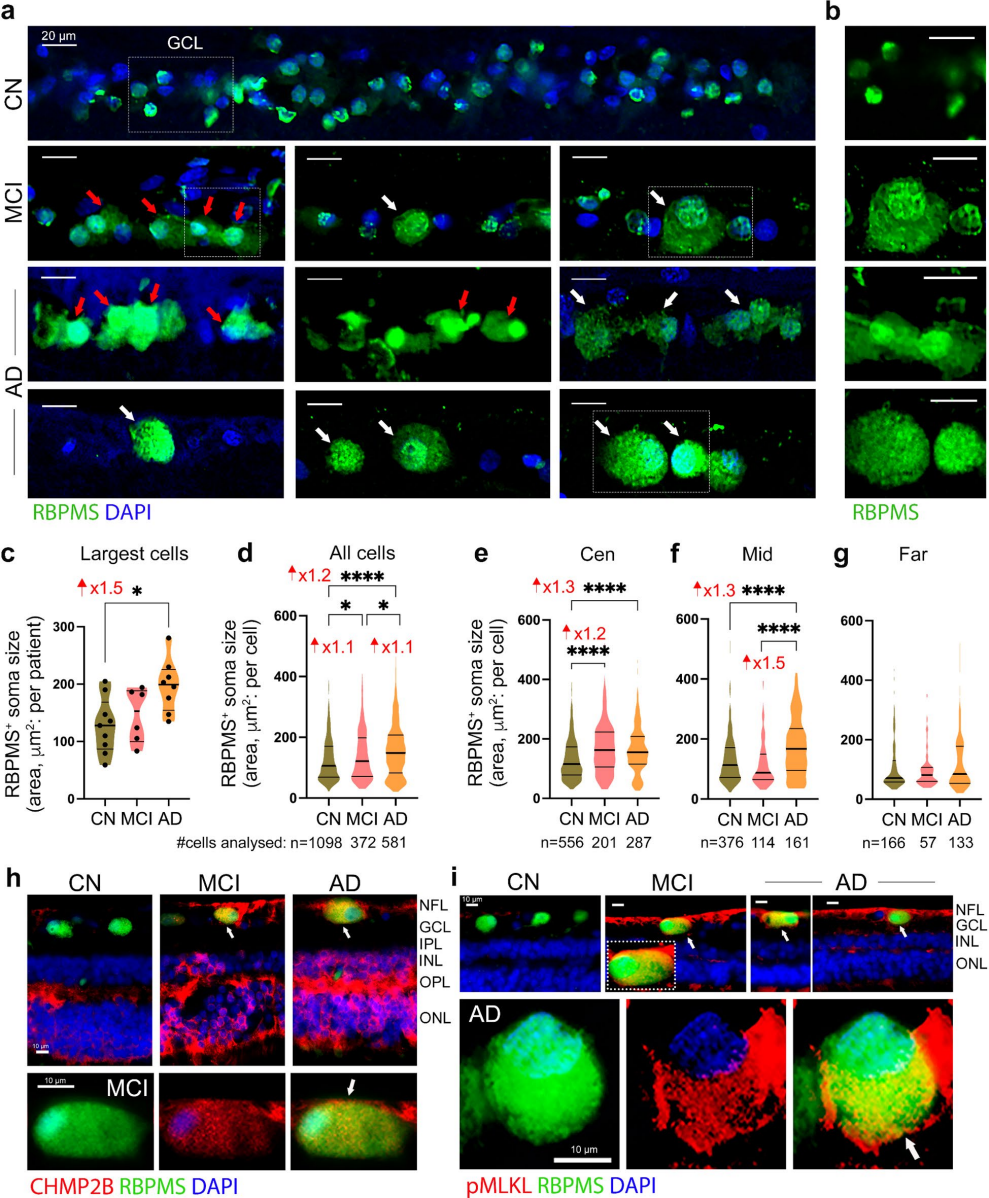
RGC hypertrophy and GVD-associated necroptosis in MCI and AD patients. (**a**) Representative microscopic images of retinal cross-section immunofluorescently labeled for RGC-specific marker RBPMS (green) and nuclei (DAPI blue), in the GCL, showing abnormal morphology of RGCs in MCI and AD retinas as compared with CN controls. Retinas from MCI and AD patients exhibit a hypertrophic cytoplasm (cell soma swelling), and abnormal morphology, including granulovacuolar vesicles degeneration (GVD)-like bodies and nucleus displacement. Red arrows point to nuclear displacement and white arrows indicate granulomatous cytoplasm. (**b**) Enlarged images of RBPMS^+^ RGCs (green channel only) in the respective diagnostic groups. Scale bars: 20 μm. (**c**) Quantitative analysis of RBPMS^+^ RGC soma cell size of the three largest cells per microscopic image, in patients with MCI and AD compared to CN controls. (**d-g**) Quantitative analysis of soma cell size for all RBPMS^+^ RGCs analyzed in total ST region (**d**), and in Cen (**e**) Mid-(**f**) and Far-(**g**) peripheral subregions, in *n* = 9 CN, *n* = 6 MCI, *n* = 10 AD; total number of cells that were analyzed are indicated below the graphs. (**h**) Representative microscopic images of retinal cross-section immunofluorescently labeled for the GVD marker, charged multivesicular body protein 2B (CHMP2B, red), RBPMS for RGCs (green), and DAPI for nuclei (blue) in CN, MCI, and AD donors. Colocalization of CHMP2B within RBPMS^+^ RGCs (yellow) is indicated by white arrows. Scale bars: 10 μm. (**i**) Representative microscopic images of retinal cross-section immunolabeled for the GVD-associated necroptotic marker, phosphorylated mixed lineage kinase domain-like (pMLKL, red), RBPMS (green), and DAPI (blue) in CN, MCI, and AD subjects. Colocalization of pMLKL within RBPMS (yellow) is indicated by white arrows. Scale bars: 10 μm. Individual data points and median, lower, and upper quartiles are shown in violin plots. *P < 0.05, ****P < 0.0001, by one-way ANOVA followed by Tukey’s post-hoc multiple comparison test. Fold changes are shown in red. GCL, Ganglion cell layer; NFL, Nerve fiber layer; IPL, Inner Plexiform Layer; INL, Inner Nuclear Layer; OPL, Outer Plexiform Layer; ONL, Outer Nuclear Layer

We next analyzed the morphological changes in RGCs of MCI and AD patients compared to CN controls (Fig. 2a, b). We found that RGCs in MCI and AD patients often exhibited a hypertrophic cell soma, with nucleus displacement, and patterns of granulovacuolar degeneration (GVD)-like bodies, as indicated by red and white arrows, respectively (Fig. 2a). Quantitative analysis of the largest RBPMS^+^ ganglion cells, across the central, mid- and far-peripheral ST retina, revealed a significant 1.5-fold enlargement of the granulomatous soma-cell area in AD patients compared to CN controls (Fig. 2c; *P* < 0.05), with no significant change observed in the MCI group. Due to the highly diverse population of RGCs [56], we also performed an extensive quantification of all identifiable RBPMS^+^ RGC soma size (a total of 2,051 cells), across the ST retina (Fig. 2d) and in the central, mid-periphery, and far-periphery subregions (Fig. 2e-g). This analysis indicated significant 1.1-1.3-fold increases of RGC-soma area in MCI and AD relative to CN (*P* < 0.05-*P* < 0.0001). RGC hypertrophy was highly significant in the central and mid-peripheral subregions (Fig. 2e, f). No significant RGC-soma area increase was observed in the far-periphery subregion (Fig. 2g). The observation of GVD-like punctate staining patterns in RBPMS^+^ RGCs of MCI and AD patients prompted us to investigate whether markers of GVD and GVD-associated activation of the necroptosis pathway (GVD-necroptosis) were present in these cells (Fig. 2h, i). Immunohistochemistry of the GVD marker, charged multivesicular body protein 2B (CHMP2B), commonly detected in brain neuronal GVDs [130], revealed prominent CHMP2B expression in RBPMS^+^ RGCs of MCI and AD patients, including in hypertrophic RGCs (Fig. 2h, white arrows). Furthermore, immunolabeling for the GVD-associated activation of necroptosis pathway (GVD-necroptosis) marker, phosphorylated mixed lineage kinase domain-like (pMLKL) [60, 61] confirmed the presence of pMLKL GVD-necroptotic proteins within RBPMS^+^ RGCs of MCI and AD patients (Fig. 2i, white arrows).

### Increased pS396-tau and oligo-tau in RGCs linked to RGC depletion

**Fig. 3:**
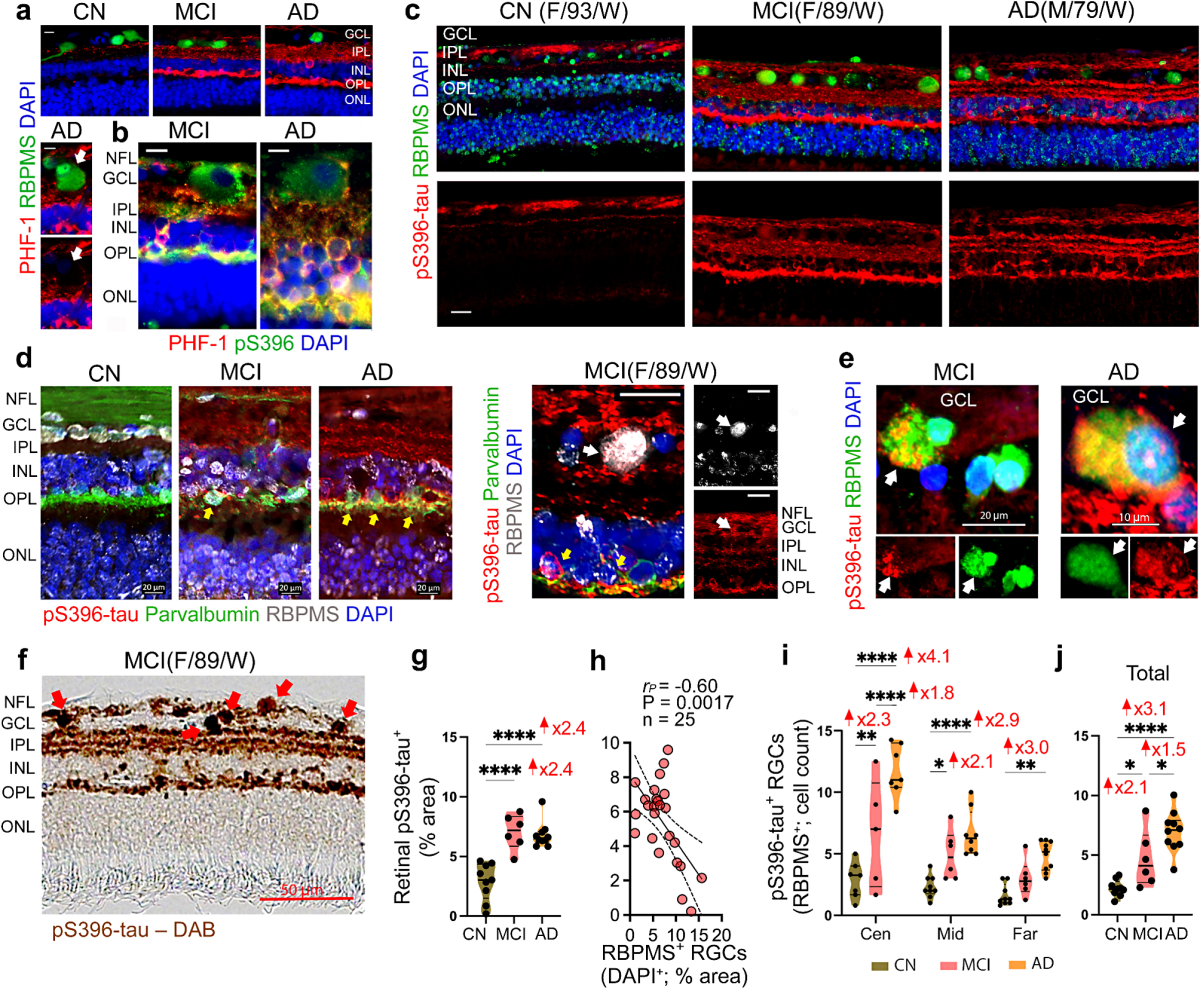
Phosphorylated tau pathology in RGCs of MCI and AD patients. (**a**) Representative microscopic images of retinal cross-sections immunolabeled for paired helical filament of tau (PHF-1, red), RBPMS (green), and DAPI for nuclei (blue) in CN, MCI, and AD subjects. Scale bars: 10 μm. PHF-tau is usually not found within RGCs of MCI and AD patients, as shown in top and bottom left panels and indicated by white arrows. (**b**) Representative microscopic images of retinal cross-sections immunofluorescently co-labeled for PHF-tau (PHF-1, red) and hyperphosphorylated (p)tau at S396 epitope (pS396-tau, green), with nuclei (DAPI, blue) in MCI and AD donors. Scale bars: 10 μm. RGCs appear to stain for pS396-tau, and not PHF-tau, as shown by cell morphology and location in the GCL, while other layers (IPL, INL, OPL) show colocalization (yellow) of PHF-tau and pS396-tau. Scale bars: 10 μm. (**c**) Representative micrographs of retinal cross-sections immunolabeled for pS396-tau (red), RGCs (RBPMS, green), and nuclei (DAPI, blue) in CN, MCI, and AD donors. Scale bars: 20 μm. (**d**) Retinal micrographs immunolabeled for RBPMS RGCs (white), pS396-tau (red), amacrine and RGCs marker - parvalbumin (green), and nuclei (DAPI, blue) in CN, MCI, and AD subjects. Colocalization of pS396-tau in parvalbumin^+^ amacrine cells (yellow arrows, left panel) and RBPMS^+^ RGCs (white arrows, middle panel) are shown. Scale bars: 20 μm. (**e**) High-magnification microscopic images depicting pS396-tau (red) accumulation in hypertrophic RBPMS^+^ RGCs (green); white arrows indicate pS396-tau-laden RGCs in MCI and AD patients (yellow for colocalization). Scale bars: 20 μm (left) and 10 μm (right). Bottom panels show separate channels for each pS396-tau and RBPMS. (**f**) A representative microscopic image of peroxidase-based staining for pS396-tau isoforms (brown) within retinal layers, and specifically, in RGCs (by morphology and location, red arrows) of an MCI patient. Scale bar: 50 μm. (**g**) Quantification of pS396-tau percent area in the ST retina (*n* = 9 CN, *n* = 6 MCI, *n* = 10 AD). (**h**) Pearson’s correlation coefficient (*r*_*P*_) analysis between retinal pS396-tau and RBPMS^+^ RGCs. (**i-j**) Cell count of pS396-tau^+^ RGCs in (**i**) Central, Mid-, and Far-peripheral retinal subregions, and (**j**) total ST retina (*n* = 19–25). Individual data points and median, lower, and upper quartiles are shown in violin plots. *P < 0.05, **P < 0.01, ****P < 0.0001, by one-way or two-way ANOVA followed by Tukey’s post-hoc multiple comparison test. Fold changes are shown in red. F, female; M, male; Age (in years); Ethnicity: W, White; NFL, Nerve fiber layer, GCL, ganglion cell layer; IPL, Inner Plexiform Layer; INL, Inner Nuclear Layer, OPL, Outer Plexiform Layer; ONL, Outer Nuclear Layer; RGC, Retinal ganglion cells

**Fig. 4:**
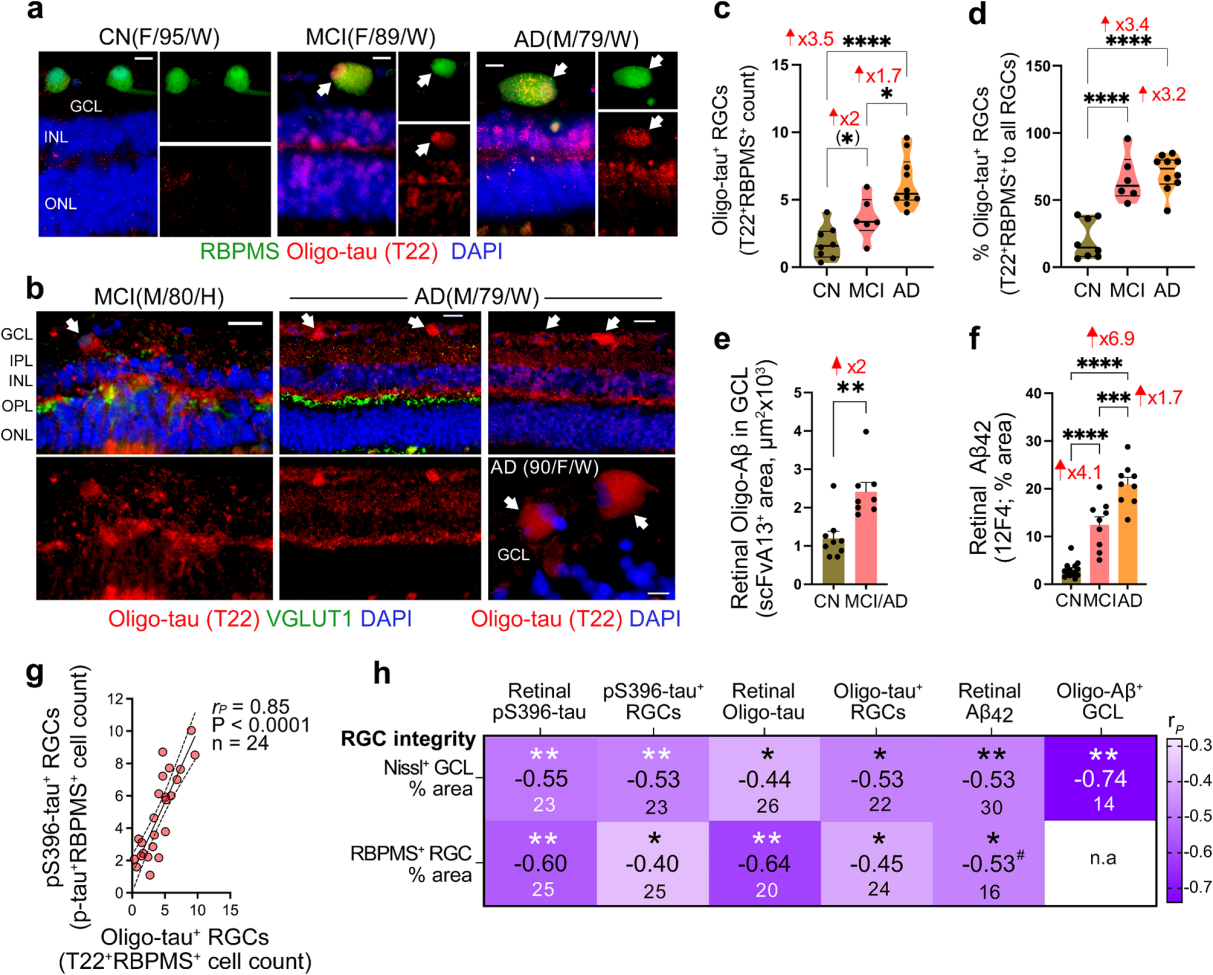
Oligomeric tau isoforms within RGCs in MCI and AD patients. (**a**) Representative retinal cross-sections immunolabeled for Oligo-tau (T22, red), RBPMS^+^ RGCs (green), and nuclei (DAPI, blue), showing Oligo-tau colocalization in RGCs (yellow, white arrows) in MCI and AD vs. CN. Scale bars: 10 μm. (**b**) Representative retinal cross-sections immunolabeled for Oligo-tau (T22^+^, red), pre-synaptic marker VGLUT1 (green), and nuclei (DAPI, blue) in MCI and AD patients, showing Oligo-tau-laden RGCs, per morphology and cell layer (white arrows). Scale bars: 20 μm. (**c**) Quantification of T22^+^ Oligo-tau RBPMS^+^ RGC count in MCI (*n* = 6) and AD (*n* = 10) patients vs. CN controls (*n* = 8). (**d**) Percent of Oligo-tau^+^ RBPMS^+^ RGC count to total RBPMS^+^ RGC count in this cohort. (**e**) Quantitative analysis of retinal Oligo-Aβ in the GCL (scFvA13^+^ immunoreactive area within β-III-tubulin^+^ neurons) in this cohort (*n* = 8 MCI/AD, *n* = 9 CN). (**f**) Quantitative analysis of retinal Aβ_42_ percent area in this cohort (*n* = 9 MCI, *n* = 9 AD, *n* = 14 CN). (**g**) Pearson’s correlation coefficient (*r*_*P*_) analysis between Oligo-tau^+^ RGC and pS396-tau^+^ RGC cell counts. (**h**) Heatmap displays Pearson’s *r*_*P*_ correlations of RGC integrity parameters (% area of RBPMS^+^ RGCs and Nissl^+^ cells in GCL) with the following abnormal tau and Aβ forms in the retina and within RGCs: retinal pS396-tau (% area), pS396-tau^+^ RGCs (count), retinal T22^+^ Oligo-tau (% area), T22^+^ Oligo-tau^+^ RGCs (% cell count), retinal 12F4^+^ Aβ_42_ (% area), and scFvA13^+^ Oligo-Aβ in the GCL (area). Large-font numbers indicate Pearson’s *r* values and lower-font numbers indicate sample size (n). Individual data points and median, lower, and upper quartiles are shown in violin plots. Bar graphs display mean ± SEM. *P < 0.05, **P < 0.01, ***P < 0.001, ****P < 0.0001, by one-way or two-way ANOVA followed by Tukey’s post-hoc multiple comparison test or unpaired Student *t*-test (in parenthesis). Statistical significance shown in the heatmap is calculated by Pearson’s correlation analyses. Fold changes are shown in red. F, female; M, male; Age (in years); Ethnicity: W, White and H, Hispanic; GCL, ganglion cell layer; IPL, Inner Plexiform Layer; INL, Inner Nuclear Layer, OPL, Outer Plexiform Layer; ONL, Outer Nuclear Layer; RGC, Retinal ganglion cells

We recently reported significant increases in abnormal tau isoforms, particularly pretangle forms such as pS396-tau and Oligo-tau, in the retina of MCI and AD patients [112]. Here, we investigated whether RGCs are vulnerable to the accumulation of these pretangle (pS396-tau and Oligo-tau) and mature tangle (PHF-tau) isoforms in early and advanced AD patients (Figs. 3 and 4; extended data in Suppl. Figure 2 and Suppl. Figure 3). First, we performed IHC analysis employing antibody combinations of RBPMS with PHF-1 (Fig. 3a) and PHF-1 with pS396 (Fig. 3b). The pS396 antibody detects tau phosphorylated at serine 396, while the PHF-1 antibody recognizes pS396- and pS404-tau in paired helical filaments. We found that PHF-tau was rarely observed within the soma of RBPMS^+^ RGCs, and most RGCs were PHF-tau negative (Fig. 3a, white arrows in lower image). In contrast, pS396-tau was frequently detected within RGCs of MCI and AD patients (Fig. 3b-e). Excluding the RGC soma, both PHF-1 and pS396 colocalized in the inner retinal layers and OPL in MCI and AD patients (Fig. 3b, yellow). Increased pS396-tau burden in the OPL, IPL, GCL, and NFL, accompanied by hypertrophic soma and reduced RBPMS^+^ RGCs, was observed in MCI and AD patients compared to CN controls (Fig. 3c). The three-parallel-string staining pattern of retinal pS396-tau in the IPL of MCI and AD patients appeared to accumulate in the neuronal dendrites of RGCs, connecting with axons of bipolar and amacrine cells. We next immunolabeled retinal cross-sections for pS396-tau in combination with RBPMS and parvalbumin, the latter being a marker of horizontal cells within the OPL and RGCs [58]. This analysis identified pS396-tau accumulation within enlarged RBPMS^+^ RGCs (white arrows) and horizontal cells (yellow arrows) of MCI and AD patients, and occasionally in RBPMS^+^ RGCs of CN individuals (Fig. 3d, e and Suppl. Figure 2a). The pS396-tau buildup within the somas of RGCs in the GCL was also evident in non-fluorescence, peroxidase-based IHC staining (Fig. 3f, red arrows).

Quantitative IHC analysis of retinal pS396-tau^+^ percent area showed highly significant 2.4-fold increases in MCI and AD patients compared to CN controls (Fig. 3g; *p* < 0.0001). Pearson’s correlation coefficient (*r*_*P*_) analysis revealed an inverse correlation between retinal pS396-tau burden and RBPMS^+^ RGC area (Fig. 3h; *r*_*P*_= -0.60 and *P* = 0.0017). Notably, pS396-tau-positive RBPMS^+^ RGC counts were significantly elevated in MCI (2.1-2.3-fold; *P* < 0.05 − 0.01) and further increased in AD (2.9-4.1-fold; *P* < 0.01-0.0001) retinas, compared to CN controls, across retinal subregions and in the total ST region (Fig. 3i, j). Increases in pS396-tau^+^ RBPMS^+^ RGC count, as well as the percentage area of pS396-tau^+^ in the GCL of MCI and AD patients, were more significant in the central ST retina (Fig. 3i and Suppl. Figure 2b, c). Elevated retinal pS396-tau was associated with a higher number of pS396-tau-laden RGCs and increased CCasp3 in the GCL (Suppl. Figure 2d, e).

Next, IHC analysis of T22^+^ Oligo-tau in the retina of MCI and AD patients compared to CN controls identified oligo-tau deposition within swollen RBPMS^+^ RGCs (Fig. 4a, white arrows) and prominent Oligo-tau staining in the GCL (Fig. 4b; T22^+^ RGCs are indicated by morphology and location with white arrows). Quantitative analysis of retinal Oligo-tau^+^ percent area confirmed the substantial 5.6- and 9.6-fold increases in MCI and AD patients, respectively, compared to CN controls (Suppl. Figure 3a; *p* < 0.01 − 0.001). Notably, both Oligo-tau^+^ RGC counts and percent Oligo-tau^+^ RGC population were significantly elevated in MCI and AD (2.0-3.5-fold; *P* < 0.05-<0.0001) compared to CN controls in the ST retina (Fig. 4c, d; extended data across retinal subregions in Suppl Fig. 3b, c). No difference in percent Oligo-tau-laden RGC population was observed between MCI and AD (Fig. 4d), whereas a significant 1.7-fold further increase in Oligo-tau-laden RGC counts was detected in AD dementia compared to the MCI group (Fig. 4c; *P* < 0.05). Similar to pS396^+^ RGCs, increases in Oligo-tau^+^ RGCs in MCI and AD were more pronounced and significant in the central subregion (Suppl Fig. 3b, c). Concomitant with abnormal tau accumulation in RGCs, we also detected significant increases in intra-RGC scFvA13^+^ Aβ oligomers and retinal 12F4^+^ Aβ_42_ burden in this cohort of MCI and AD patients versus CN controls (Fig. 4e, f). Interestingly, we found a very strong correlation between pS396- and Oligo-tau laden RGCs (Fig. 4g; *r*_*P*_=0.85 and *P* > 0.0001).

We further investigated the interrelations between retinal abnormal tau and Aβ burden, including in RGCs, and RGC integrity, as assessed by Nissl^+^ in GCL and RBPMS^+^ RGCs percent areas (Fig. 4h; extended data in Suppl. Figure 3d-g). Pearson’s (*r*) correlation analysis showed inverse correlations between RGC integrity and retinal Aβ_42_ burden (Fig. 4h and Suppl. Figure 3e; *r*_*P*_= -0.53 and *P* = 0.033), and intraneuronal Oligo-Aβ in GCL (Fig. 4h and Suppl. Figure 3f; *r*_*P*_= -0.74 and *P* = 0.0022). Notably, we found multiple inverse associations between RGC integrity and pS396-tau and Oligo-tau burden in the retina, and specifically in RGCs (Fig. 4h and Suppl. Figure 3d, g; *r*_*P*_= -0.40-(-0.64) and *P* < 0.05 − 0.01). These data suggest that elevated levels of abnormal pretangle tau forms in the retina, including within RGCs, are linked to RGC loss.

### Retinal pS396-tau and oligo-tau laden ganglion cells correlate with AD status

**Fig. 5:**
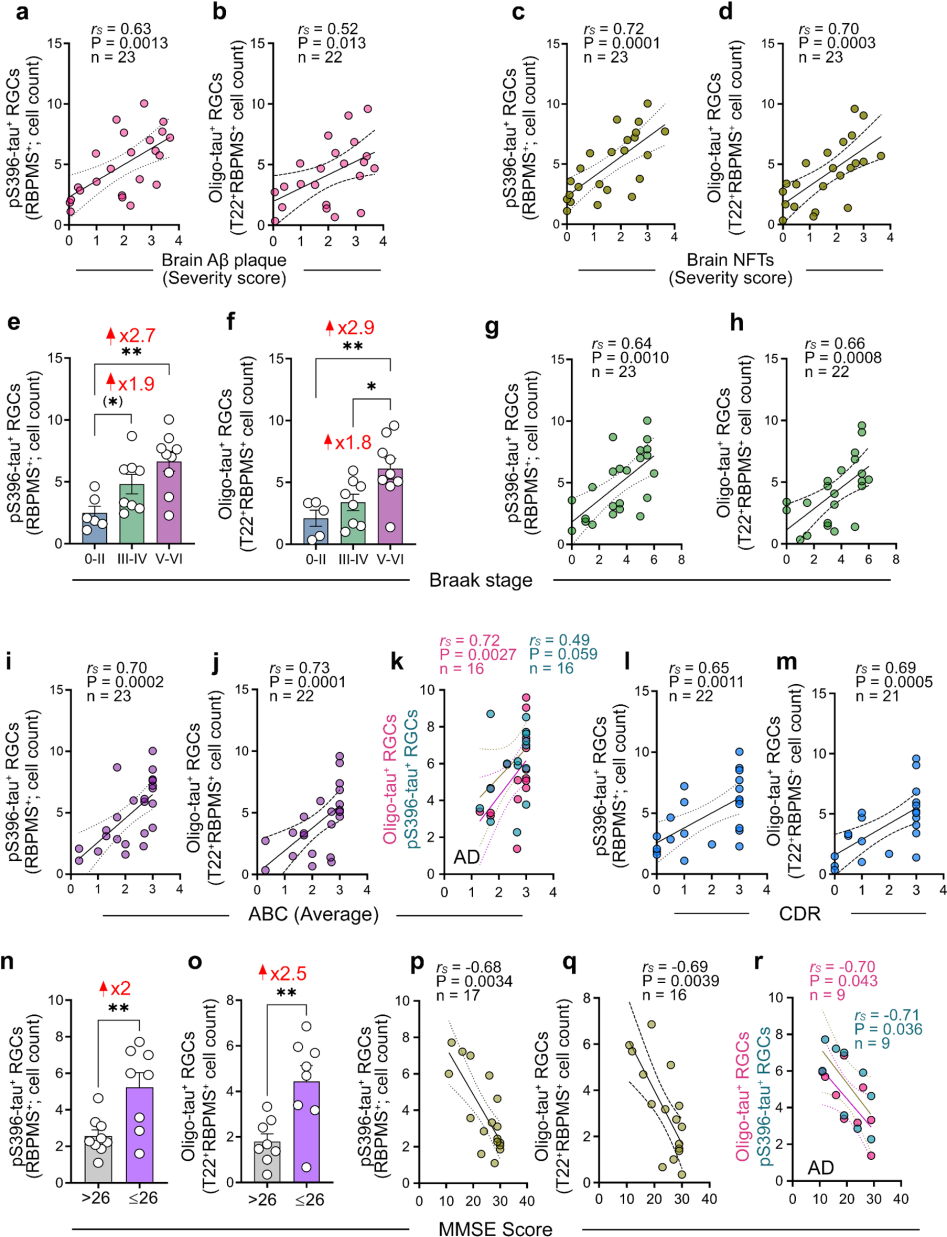
Interactions of Oligo-tau and pS396-tau-laden RGCs with brain pathology and cognitive status. (**a-d**) Spearman’s rank correlation coefficient (*r*_*S*_) analyses of pS396-tau^+^ RGCs (count) or T22^+^ Oligo-tau^+^ RGCs (count) with (**a**, **b**) brain Aβ plaque severity scores or (**c**, **d**) brain neurofibrillary tangles (NFTs) severity scores. (**e**, **f**) Quantitative pS396-tau^+^ RGC and Oligo-tau^+^ RGC cell counts per Braak stage stratification (*n* = 22–23). (**g**, **h**) Spearman’s *r*_*S*_ correlations of (**g**) pS396-tau^+^ RGCs (count) or (**h**) Oligo-tau^+^ RGCs (count) with the Braak stage. (**i-m**) Spearman’s *r*_*S*_ correlations of pS396-tau^+^ RGCs (count) or Oligo-tau^+^ RGCs (count) with (**i-k**) average brain ABC scores (**k** for MCI due-to-AD and AD dementia patients only), and with (**l-m**) clinical dementia rating (CDR) scores. (**n**, **o**) Quantitative pS396-tau^+^ RGC and Oligo-tau^+^ RGC cell counts when patients are stratified according to 26-score cutoff on the mini-mental state examination (MMSE; *n* = 16–17). (**p-r**) Spearman’s *r*_*S*_ correlations between pS396-tau^+^ RGCs or Oligo-tau^+^ RGCs cell counts and MMSE scores (**r** for MCI due-to-AD and AD dementia patients only). Bar graphs display individual data points and mean ± SEM. *P < 0.05, **P < 0.01, by one-way ANOVA followed by Tukey’s post-hoc multiple comparison test. Two group comparison is determined by unpaired Student *t*-test. ABC scores comprise of mean grades for: (**A**) Aβ plaque score modified from Thal, (**B**) NFT stage modified from Braak, and (**C**) neuritic plaque score modified from CERAD

We further determined the potential relationship between pS396-tau^+^ or Oligo-tau^+^ RBPMS^+^ RGCs or RGC integrity, and the severity of AD-related brain pathology (Fig. 5a-k; extended data for correlations across retinal subregions in Table 2). Spearman’s rank correlation coefficient (*r*_*S*_) analyses revealed that pS396-tau^+^ and Oligo-tau^+^ RGC counts moderate-to-strongly associated with brain Aβ-plaque burden (Fig. 5a, b; *r*_*S*_=0.52–0.63, *P* = 0.013 − 0.0013) and NFT severity scores (Fig. 5c, d; *r*_*S*_=0.70–0.72, *P* = 0.0003 − 0.0001). Stratifying patients based on Braak stage severity showed significant 1.9-2.7-fold increases in pS396-tau^+^ RBPMS^+^ RGCs in the high (V-VI) and, to a lesser extent, the intermediate (III-IV) Braak stage groups compared to the low (0-II) group (Fig. 5e; *P* < 0.05 − 0.01). Similarly, Oligo-tau^+^ RBPMS^+^ RGCs were 2.9-fold elevated in the high (V-VI) Braak stage group compared to the low (0-II) group (Fig. 5f; *P* < 0.05 − 0.01). Both pS396-tau and Oligo-tau laden RGC counts were strongly correlated with Braak stage (Fig. 5g, h; *r* = 0.64–0.66, *P* = 0.001 − 0.0008). In addition, pS396-tau and Oligo-tau laden RGC counts were strongly correlated with ABC disease severity scores (Fig. 5i-k; *r* = 0.70–0.73, *P* = 0.0002 − 0.0001), with strong associations remaining when analyzed for MCI (due to AD) and AD patients only (Fig. 5k). No significant correlations were detected between RGC integrity parameters (RBPMS^+^ RGC count, Nissl^+^ in GCL % area) and any of the AD-related brain pathology scores (Table 2). Interestingly, analyses per retinal subregions detected stronger associations to AD-brain pathology for *central* retinal pS396-tau^+^ RGCs and *mid-peripheral* Oligo-tau^+^ RGCs (Table 2), with a very strong association between mid-peripheral Oligo-tau^+^ RGCs and brain NFTs (*r*_*S*_=0.81, *P* < 0.0001).

**Table 2:** Correlations between RGC parameters and brain pathology

	Brain Aβ Plaque (score)		Brain NFTs (score)		BRAAK (stage)		ABC (score)	
	*N* = 17–23		*N* = 14–23		*N* = 17–23		*N* = 14–23	
	*r*_S_	*P*	*r*_S_	*P*	*r*_S_	*P*	*r*_S_	*P*
**RBPMS** ^**+**^ **RGCs (count)**								
Total	-0.17	0.44	-0.20	0.36	-0.16	0.46	-0.17	0.44
Central	-0.14	0.58	-0.17	0.50	-0.15	0.56	-0.17	0.51
Mid-periphery	-0.32	0.16	-0.33	0.14	-0.35	0.12	-0.40	0.073
Far-periphery	-0.25	0.24	-0.30	0.17	-0.28	0.20	-0.24	0.27
**Nissl** ^**+**^ **in GCL (% area)**								
Total	-0.14	0.56	-0.10	0.66	-0.019	0.93	-0.085	0.71
Central	-0.074	0.78	-0.23	0.38	-0.20	0.43	-0.17	0.50
Mid-periphery	-0.13	0.56	-0.092	0.69	-0.054	0.82	-0.11	0.63
Far-periphery	-0.35	0.13	-0.025	0.92	0.0084	0.97	-0.17	0.48
**pS396-tau** ^**+**^ **RGCs (count)**								
Total	**0.63**	0.0013**	**0.72**	0.0001***	**0.64**	0.0010**	**0.70**	0.0002***
Central	**0.65**	0.0034**	**0.69**	0.0014**	**0.68**	0.0020**	**0.72**	0.0008***
Mid-periphery	**0.51**	0.019*	**0.74**	0.0001***	**0.64**	0.0018**	**0.61**	0.0032**
Far-periphery	**0.58**	0.0039**	**0.72**	0.0001***	**0.63**	0.0012**	**0.68**	0.0003***
**Oligo-tau** ^**+**^ **RGCs (count)**								
Total	**0.52**	0.013*	**0.70**	0.0003***	**0.66**	0.0008***	**0.73**	0.0001***
Central	0.40	0.14	**0.67**	0.0073**	**0.58**	0.026*	**0.59**	0.022*
Mid-periphery	**0.65**	0.0035**	**0.81**	< 0.0001****	**0.78**	0.0001***	**0.79**	< 0.0001****
Far-periphery	0.26	0.24	**0.50**	0.017*	**0.47**	0.027*	**0.47**	0.029*

Spearman’s rank correlation analyses: P and *r*_*S*_ -values determine the statistical significance and strength of each pairwise association between retinal RGC marker and brain pathology. P and *r*_*S*_ -values presented in bold type with asterisk(s) are statistically significant (< 0.05). Aβ, amyloid beta-protein; NFTs, neurofibrillary tangles. ABC scores comprise of mean grades for: (A) Aβ plaque score modified from Thal, (B) NFT stage modified from Braak, and (C) neuritic plaque score modified from CERAD

Finally, we evaluated the associations between tau-laden RGC counts, RGC integrity parameters, and cognitive status, as measured by clinical dementia rating (CDR) and mini-mental state examination (MMSE) tests (Fig. 5l-r; extended data across retinal subregions in Table 3). No significant correlations were detected between RGC integrity parameters in the total ST retina and cognitive scores, whereas moderate-to-strong associations were observed in specific retinal subregions (Table 3). Strong correlations were found between both pS396-tau and Oligo-tau laden RBPMS^+^ RGCs and CDR scores (Fig. 5l, m; *r* = 0.65–0.69, *P* = 0.0011 − 0.0005), for the total ST retina and most subregions (Table 3). Stratifying patients based on a MMSE cut-off score of 26, which has been reported to have high sensitivity and specificity for detecting dementia [90], was utilized in our cohort. This analysis showed a significant 2-2.5-fold increase in pS396-tau^+^ and Oligo-tau^+^ RGCs in the MMSE ≤ 26 group compared to the MMSE > 26 group (Fig. 5n, o; *P* < 0.01). Strong inverse correlations were observed between pS396-tau^+^ or Oligo-tau^+^ RGCs and MMSE scores (Fig. 5p-r; *r*=-0.68-(-0.69), *P* = 0.0039 − 0.0034), including analyses restricted to MCI (due to AD) and AD patients (Fig. 5r), for both the total retina and across retinal subregions (Table 3; extended correlations for MCI and AD patients only in Suppl. Table 3).

**Table 3:** Correlations between RGC parameters and the cognitive status

	CDR (score)		MMSE (score)	
	*N* = 13–22		*N* = 10–18	
	*r*_S_	*P*	*r*_S_	*P*
**RBPMS** ^**+**^ **RGCs (count)**				
Total	-0.33	0.13	0.26	0.31
Central	-0.06	0.83	0.15	0.59
Mid-periphery	-0.43	0.056	0.46	0.065
Far- periphery	**-0.51**	0.016*	0.32	0.20
**Nissl** ^**+**^ **in GCL (% area)**				
Total	-0.40	0.080	0.48	0.070
Central	**-0.52**	0.041*	0.28	0.35
Mid-periphery	-0.35	0.13	0.58*	0.024*
Far-periphery	-0.34	0.16	0.64*	0.012*
**pS396-tau** ^**+**^ **RGCs (count)**				
Total	**0.65**	0.0011**	**-0.68**	0.0034**
Central	**0.61**	0.011*	**-0.62**	0.015*
Mid-periphery	**0.63**	0.0030**	**-0.58**	0.017*
Far-periphery	**0.65**	0.0011**	**-0.54**	0.026*
**Oligo-tau** ^**+**^ **RGCs (count)**				
Total	**0.69**	0.0005***	**-0.69**	0.0039**
Central	**0.68**	0.0096**	**-0.67**	0.028*
Mid-periphery	**0.64**	0.0042**	**-0.79**	0.0019**
Far-periphery	0.39	0.0837	-0.41	0.1180

Spearman’s rank correlation analyses: P and *r*_*S*_ -values determine the statistical significance and strength of each pairwise association between retinal RGC marker and the cognitive function score. P and *r*_*S*_ -values presented in bold type with asterisk(s) are statistically significant (< 0.05). CDR, Clinical Dementia Rating; MMSE, Mini-Mental State Examination

## Discussion

In this study, we present the first evidence of pathogenic tau inclusions, specifically pS396-tau and Oligo-tau, within RBPMS-positive RGCs, concomitant with ganglion cell loss (~ 50%) in the retinas of donor patients with MCI (due to AD) and AD dementia. Increases in these pretangle isoform-containing RGCs in both MCI and AD patients were accompanied by hypertrophic RGC soma and nuclei displacement, as well as elevated apoptotic cell markers (~ 30%) and the expression of GVD (CHPM2B) and GVD-associated necroptotic (pMLKL) markers in RGCs. We revealed a very strong association between the two pretangle tau forms in RGCs. Notably, we found moderate to strong associations between RGC loss and retinal pS396-tau or Oligo-tau burden, including tau-laden RGCs. Retinal Aβ_42_ and intra-RGC Aβ oligomers also associated with RGC reduction, suggesting a link between retinal tau and amyloid pathologies and ganglion cell integrity in AD. Importantly, our data indicated tight correlations between pS396-tau or Oligo-tau-containing RGCs and respective brain pathology, disease stage, and cognitive status. The most consistent and strong correlations with brain pathology and cognition were found for pS396-tau-laden RGCs in the central/mid-peripheral subregions, and for Oligo-tau-laden RGCs in the mid-periphery. Overall, our findings suggest that abnormal tau isoforms accumulate within RBPMS^+^ RGCs in patients with MCI and AD, potentially contributing to early and pronounced RGC loss and showing a strong correlation with disease severity.

Among the RGC populations, midget cells projecting to the parvocellular (P-cell) layers of the lateral geniculate nucleus (LGN) and parasol cells projecting to the magnocellular (M-cell) layers of the LGN serve as two distinct visual pathways that process color and low spatial frequency contrast vision, respectively [72]. In AD patients, abnormalities in color vision, eye movement, contrast sensitivity, and visual integration have been detected early in disease progression [33, 38, 47, 75, 81, 105]. Therefore, fluctuations in color perception and abnormal contrast sensitivity in AD patients may be attributed to damage and loss of these RGC types, in addition to the involvement of horizontal and amacrine neurons. Here, the analysis of the RBPMS marker, a conserved RNA binding protein with a single RNA recognition motif expressed in RGCs of humans and animal models [87, 94], facilitates differentiation from other retinal cells [66, 95, 102], further validating our findings in RGCs. Our current analysis of RGC integrity in the superior temporal retina indicates marked decreases in RBPMS^+^ RGC counts or immunoreactive area (by 47–55% in MCI and 46–50% in AD) compared to CN controls, with similar degrees of decreases observed in GCL Nissl^+^ neurons (by 56% in MCI and 55% in AD patients). These results are consistent with previous studies reporting significant reductions in RGCs and GCL thickness in AD patients compared to control subjects [8, 16–18, 109].

Specifically, the RBPMS^+^ RGC count per retinal subregion indicated a 46% loss in MCI and a 57% loss in AD in the mid-periphery, as well as a 62% loss in MCI and a 45% loss in AD in the far periphery. Similarly, a study by Blanks et al., described GCL neuronal loss in AD as most pronounced in the superior and inferior quadrants, ranging from 40 to 49% throughout the mid-peripheral subregions, and reaching 50–59% in the far-peripheral retina of AD patients [16]. These peripheral retinal subregions, which have anatomically fewer ganglion cells and a thinner nerve fiber layer, appear more vulnerable to RGC loss in AD, potentially due to a higher density of abnormal Aβ and tau species (e.g., Aβ_42_, Aβ oligomers, PHF-tau, pS396-tau, and pS202/T205-tau), and microgliosis [63, 64, 73, 112]. Interestingly, whereas the total and mid-peripheral ST retina consistently demonstrated significant and similar RGC reductions in both MCI and AD patients, as shown by the GCL Nissl^+^ area and RBPMS^+^ RGC count analyses, non-significant trends were noted for the far and central subregions, respectively. These differences may be due to variations in the types of analysis and staining patterns.

Importantly, the loss of RGCs in MCI and AD patients may explain previous reports of visual dysfunctions in AD, specifically impaired color vision, low spatial frequency contrast, and motion perception [38], which can be attributed, at least in part, to the loss of M-cell and P-cell RGCs. However, the retinal regions examined here align with the superior-temporal arcade along the temporal vessels, primarily containing axons from magnocellular pathway RGCs rather than those originating from macular areas. Consequently, the substantial RGC loss observed in these regions is unlikely to significantly affect fine or central vision provided by the macula. Having said that, Frisen and Quigley [36], in a landmark article, showed that even a 50% loss of optic nerve axons did not usually cause a diminution of visual acuity. Their histopathological study concluded that there are “extra axons” that represent redundancy. This is in close alignment with a study by Tenhula et al. [120], demonstrating histopathological counts versus loss of vision. More accurately, the “extra axons” lost probably provide for better contrast sensitivity and, indeed, AD patients suffer losses of contrast sensitivity [4, 38, 101, 104]. Subjectively, RGC losses of this magnitude are perhaps not sufficient to be described in routine neurological or ophthalmological examinations. Furthermore, many of the lost axons originate from magnocellular RGCs, which mediate low spatial frequency contrast sensitivity and motion perception. These deficits, though not consciously apparent to patients, can be objectively demonstrated through proper testing. As it relates to another functional impairment, a previous study of postmortem AD retinas identified a reduction in melanopsin retinal ganglion cells (mRGCs), intrinsically photosensitive cells that contribute to the photoentrainment of circadian rhythms, potentially explaining the sleep disturbances observed in these patients [70].

In the brains of AD patients, the increase in hyperphosphorylated tau isoforms has been shown to lead to tau aggregation, oligomerization, propagation, and NFT formation, ultimately causing neuronal dysfunction and degeneration [93, 119]. Previous studies have detected intracellular pretangles and mature tangles in the retinas of AD patients [28, 30, 40, 45, 46, 63, 89, 112, 129]. Recently, we also identified tau oligomers and citrullinated-tau, along with other tau isoforms, in the retinas of MCI and AD patients. Notably, both pS396-tau and oligomeric-tau forms were frequently observed within the GCL, with significant increases in MCI and AD patients [112]. The pS396-tau and Oligo-tau isoforms are increased in the AD brains and are linked to neuronal cell loss and Braak stage severity [9, 25, 39, 96, 104, 117, 133]. Here, we found a specific buildup of these pathological tau isoforms within RGCs of MCI and AD patients, demonstrating their very strong inter-relations and connection with RGC integrity, and entailing similar links between tauopathy and neurodegeneration in the retina, as observed in the brain.

In this study, we detected higher numbers of RBPMS^+^ RGCs containing pS396-tau and Oligo-tau in patients with AD dementia and those at the earliest stages of functional impairment (MCI due to AD). These pathogenic tau-laden RGC counts were higher in AD patients compared to MCI patients, suggesting that more RGCs are affected by pS396-tau and Oligo-tau as the disease progresses. Our data on pS396-tau and Oligo-tau laden RGCs across retinal subregions indicated that the most significant and substantial changes were detected in the central and mid-peripheral ST retina, with less pronounced changes in the far periphery. This could be attributed to the density of RGCs in each subregion, as there are up to eight layers of ganglion cells in the central subregion and only one or two layers, with space between them, in the far periphery [56]. Hence, there is a higher probability that RGCs in the central/middle subregions are impacted by pS396-tau and Oligo-tau compared to those in the far-peripheral retina. These findings may guide potential future in vivo imaging of tau-positive RGCs in the central and mid-peripheral ST retina for the early detection of AD.

In both fluorescent and peroxidase-based staining methods, we observed a three-parallel-string staining pattern of retinal pS396-tau in the IPL of MCI and AD patients. Consistent with retinal neuroanatomy, these findings suggest that pS396-tau accumulates within the neuronal dendrites of RGCs, which connect with the axons of bipolar and amacrine cells. These tau aggregates in synaptic-rich regions may interfere with information transmission and could help explain the decrease in contrast sensitivity observed in MCI and AD patients. Moreover, in MCI and AD patients, both pS396-tau and Oligo-tau isoforms were observed in the OPL, the former was specifically found in parvalbumin^+^ horizontal cells. A recent study suggested that pS202/T205-tau (AT8^+^) spreads from the OPL to the IPL/GCL in the AD retina [129]. The patterns of retinal pS396-tau and Oligo-tau accumulation in CN subjects and MCI/AD patients (predominately in the IPL/OPL), merit further investigation to understand how pathogenic tau spreads across retinal layers and neuronal processes during AD progression.

Beyond the moderate-to-strong inverse correlations between RGC integrity and pS396-tau^+^ or Oligo-tau^+^ RGC counts, as well as retinal pS396-tau or Oligo-tau loads, our analysis showed a strong negative association between RGC reduction and intra-RGC Aβ oligomers, suggesting their substantial and detrimental effects on RGC degeneration. These retinal findings in AD are consistent with similar reports connecting elevated Aβ and tau oligomers with neuronal loss in AD brains [3, 9, 71, 86, 109, 123]. Notably, the levels of retinal Aβ_42_ also correlated with the extent of pS396-tau or Oligo-tau-laden RGCs (not shown; *r*_*P*_=0.69 and *r*_*P*_=0.67, respectively; *P* = 0.003), as well as RGC integrity, suggesting that retinal Aβ may drive RGC tauopathy and degeneration, similar to the interactions between Aβ and the spread of tau in neurons of AD brains [18, 134].

Levels of the early apoptotic marker, cleaved caspase 3 [124], have been shown to be elevated in AD brains, with a high degree of colocalization to neurofibrillary tangles within neurons [39, 116]. In the current study, we observed increased CCasp3 expression in GCL cells, specifically in ~ 30% of RBPMS^+^ RGCs in AD dementia patients, but not in MCI patients compared to cognitively normal controls. This is consistent with previous studies showing increased CCasp3^+^/Tuj1^+^ RGCs [40] and overall retinal CCasp3 expression [64] in AD patients compared to controls. The elevated expression of retinal CCasp3 in RGCs, along with significant interactions with pS396-tau and Oligo-tau-laden RGCs, suggests that these pathogenic tau forms may trigger apoptotic RGC death in a later disease stage.

RGCs are highly diverse, consisting of multiple subtypes that exhibit a range of morphological and physiological characteristics, including variations in soma and cell body size [56]. In this study, we observed that RGCs in aged CN individuals predominantly had small-sized somas, with a minority of cells exhibiting large, round somas. In contrast, a higher number of RGCs in MCI and AD patients appeared swollen, with enlarged somas and displaced nuclei, particularly in those containing pS396-tau or Oligo-tau inclusions. Notably, RBPMS^+^ RGCs in these patients exhibited granulovacuolar degeneration (GVD) bodies marked by CHMP2B [130] and expressed the GVD-necroptotic marker pMLKL [61], suggesting that RGCs in early and advanced AD patients undergo cell death via GVD-mediated necroptosis. To the best of our knowledge, this is the first demonstration of hypertrophic RGCs expressing GVD bodies and GVD-necroptotic markers in MCI and AD patients. This abnormal RGC phenotype is characteristic of neurons exhibiting GVD-associated activation of the necroptosis pathway, indicated by phosphorylated MLKL-positive GVD bodies [60, 130]. This process has been observed in the brains of individuals with preclinical AD and AD dementia [62, 64, 121]. The morphology and process of necroptotic cells are characterized by compromised plasma membrane integrity, organelle and cell enlargement, chromatin fragmentation, and eventual cell lysis [92, 133]. Moreover, studies have indicated that necroptosis is involved in AD brain pathology and is closely linked to tau pathology and Braak stage progression [11, 21, 49, 64], with recent research showing that p-tau contributes to neuronal death by inducing necroptosis and inflammation [29]. In this study, the abnormal phenotype of RGCs, particularly in those with pS396-tau or Oligo-tau accumulation, may indicate necroptotic cell death in the RGCs of AD retinas. Future studies are needed to determine the predominant mechanism of retinal ganglion cell death and the role of pathogenic tau in apoptotic and necroptotic RGC loss in AD.

Our analysis of the potential connections between pS396-tau or Oligo-tau-containing RGCs and disease status indicates strong associations between both pretangle tau forms in RGCs and the following brain pathologies: Aβ plaques, NFTs, Braak stage, and ABC neuropathic changes. However, RGC counts alone did not correlate with these AD brain parameters. These data suggest that tauopathy-laden RGCs may represent the link between retinal neuronal injury and brain AD pathology and disease progression. Importantly, our data indicate that pS396-tau and Oligo-tau-containing RGC counts strongly correlate with cognitive status, as measured by the CDR and MMSE scores. While the CDR is a test that assess cognitive, behavioral, and functional performance associated with AD, the MMSE test evaluates cerebral competency, comprehension, and communication. We believe the strong correlations between pS396-tau^+^ or Oligo-tau^+^ RBPMS^+^ RGCs and disease parameters primarily reflect the accumulation of these tauopathy forms in the retina of AD patients. While RGC loss may have contributed modestly to these correlations, it does not independently predict tau or Aβ pathology in the brain, nor their Braak staging or ABC scoring. While cell loss is a significant feature of AD, it often does not directly correlate with the severity of brain Aβ and tau pathology. However, the observed correlations between RGC loss (in specific retinal subregions) and cognitive score (CDR, MMSE), suggest a potential link between retinal ganglion cell death and cognitive function in AD, as in the brain.

These findings highlight that a future retinal imaging approach, which reliably measures RGC-containing tauopathy in ST central or mid-peripheral subregions holds potential as a more robust biomarker, compared to RGC integrity or retinal tauopathy alone, for imaging AD progression and monitoring disease status, in both early and advanced AD. In the clinical setting, the GCL layer is assessed by OCT [38, 76, 78, 113], and apoptotic RGCs can be imaged using the detection of apoptotic retinal cells (DARC) method [10, 27]. More specific RGC changes could be detectable in the inner retina using high-resolution imaging systems, such as AO-OCT. Imaging RGCs, combined with future tauopathy tracers, could serve as a non-invasive biomarker for early AD diagnosis and monitoring of disease progression. This approach would be immensely valuable in future trials evaluating new treatments for AD.

We acknowledge several limitations of this study. As a cross-sectional, case-control study, our focus was primarily on group stratification and correlations, so caution must be exercised before implicating cause-and-effect conclusions. Moreover, the lack of clinical information on visual system-related symptoms limits our ability to assess potential connections between pS396-tau^+^ or Oligo-tau^+^ RBPMS^+^ RGCs and various manifestations of visual dysfunction. This highlights the need for future studies to explore the relationships between tauopathy-laden RGCs, RGC loss, and ocular outcomes in patients. Additionally, larger and more diverse populations are needed to validate these findings and to compare RGC susceptibility with that of other retinal cell types in relation to AD processes.

## Conclusion

In summary, this study provides the first evidence of RGCs laden with abnormal tau inclusions, including pS396-tau and oligomeric tau, in early (MCI) and AD dementia patients, with clear indications of increased RGC vulnerability. RBPMS-positive RGCs containing pS396-tau or Oligo-tau were increased in MCI and AD patients, and these cells exhibited hypertrophic cell somas as well as apoptotic and GVD/GVD-necroptotic markers. These findings correlated with reduced RGC counts, suggesting that these tau pathologies may contribute to ganglion cell death in AD. Notably, strong correlations were found between pS396-tau and Oligo-tau laden RGCs and brain AD pathology, disease stage, and cognitive status. This study highlights the potential of imaging tau-laden RGCs as a non-invasive biomarker for early AD diagnosis and monitoring disease progression. However, further research is needed to more definitively establish these connections.

## Electronic supplementary material

Below is the link to the electronic supplementary material.

Supplementary Material 1

## Data Availability

The raw datasets analyzed during the current study are available from the corresponding author on reasonable request.

## Abbreviations

A: Amyloid
Ab: Antibody
ABC: Amyloid/Braak/CERAD score
AD: Alzheimer’s disease
ADRC: Alzheimer’s disease research center
Aβ: Amyloidβ–protein
ANOVA: Analysis of variance
B: Brain
Cen (C): Central retina
CHMP2B: charged multivesicular body protein 2B
CCasp3: Cleaved caspase 3
CDR: Clinical Dementia Rating
CN: Cognitively normal
DAB: 3,3′–Diaminobenzidine
F: Female
Far (F): Far–peripheral retina
scFvA13: Single–chain Fv fragment A13 recognizing intraneuronal Aβ oligomers
GCL: Ganglion cell layer
GVD: granulovacuolar degeneration
H: Hispanic
IHC: Immunohistochemistry
INL: Inner nuclear layer
IPL: Inner plexiform layer
IR area: Immunoreactive area
M: Male
mAb: Monoclonal antibody
Mid (M): Middle–peripheral retina
MCI: Mild Cognitive Impairment
MMSE: Mini–mental state examination
mRGC: Melanopsin Retinal Ganglion Cell
NDRI: National disease research interchange
NFL: Nerve fiber layer
NFT: Neurofibrillary tangle
NT: Neuropil thread
OD: Optic disc
Oligo: Aβ–Oligomeric amyloidβ–protein
Oligo: tau–Oligomeric tau
ONL: Outer nuclear layer
OPL: Outer plexiform layer
pAb: Polyclonal antibody
PHF: Paired Helical Filaments
Phospho: MLKL–Phosphorylated Mixed Lineage Kinase Domain–Like
PMI: Postmortem interval
p: tau–Hyperphosphorylated tau
RBPMS: Ribonucleic acid binding protein with multiple splicing
RGC: Retinal Ganglion Cell(s)
Serine 396: S396
SD: Standard deviation
SEM: Standard error of the mean
ST: Superior temporal
VGLUT: 1–Vesicular glutamate transporter 1
W: White

## References

[1] (2024) 2024 Alzheimer’s disease facts and figures. Alzheimers Dement 20: 3708–3821 10.1002/alz.13809 10.1002/alz.13809 PMC11095490 38689398

[2] Alexandrov PN, Pogue A, Bhattacharjee S, Lukiw WJ (2011) Retinal amyloid peptides and complement factor H in transgenic models of Alzheimer’s disease. NeuroReport 22(12):623–627. 10.1097/WNR.0b013e3283497334 21734608 10.1097/WNR.0b013e3283497334 PMC3719862

[3] Arendt T, Bruckner MK, Morawski M, Jager C, Gertz HJ (2015) Early neurone loss in Alzheimer’s disease: cortical or subcortical? Acta Neuropathol Commun 3:10. 10.1186/s40478-015-0187-1 25853173 10.1186/s40478-015-0187-1 PMC4359478

[4] Armstrong R, Kergoat H (2015) Oculo-visual changes and clinical considerations affecting older patients with dementia. Ophthalmic Physiol Opt 35(4):352–376. 10.1111/opo.12220 26094831 10.1111/opo.12220

[5] Asanad S, Fantini M, Sultan W, Nassisi M, Felix CM, Wu J, Karanjia R, Ross-Cisneros FN, Sagare AP, Zlokovic BV et al (2020) Retinal nerve fiber layer thickness predicts CSF amyloid/tau before cognitive decline. PLoS ONE 15:e0232785. 10.1371/journal.pone.0232785 32469871 10.1371/journal.pone.0232785 PMC7259639

[6] Asanad S, Felix CM, Fantini M, Harrington MG, Sadun AA, Karanjia R (2021) Retinal ganglion cell dysfunction in preclinical Alzheimer’s disease: an electrophysiologic biomarker signature. Sci Rep 11:6344. 10.1038/s41598-021-85010-1 33737516 10.1038/s41598-021-85010-1 PMC7973731

[7] Asanad S, Ross-Cisneros FN, Nassisi M, Barron E, Karanjia R, Sadun AA (2019) The retina in Alzheimer’s Disease: histomorphometric analysis of an ophthalmologic biomarker. Invest Ophthalmol Vis Sci 60:1491–1500. 10.1167/iovs.18-25966 30973577 10.1167/iovs.18-25966 PMC6892387

[8] Ashraf G, McGuinness M, Khan MA, Obtinalla C, Hadoux X, van Wijngaarden P (2023) Retinal imaging biomarkers of Alzheimer’s disease: a systematic review and meta-analysis of studies using brain amyloid beta status for case definition. Alzheimers Dement (Amst) 15(2):e12421. 10.1002/dad2.12421 10.1002/dad2.12421 PMC10210353 37250908

[9] Baker-Nigh A, Vahedi S, Davis EG, Weintraub S, Bigio EH, Klein WL, Geula C (2015) Neuronal amyloid-beta accumulation within cholinergic basal forebrain in ageing and Alzheimer’s disease. Brain 138(6):1722–1737. 10.1093/brain/awv024 25732182 10.1093/brain/awv024 PMC4542619

[10] Balendra SI, Normando EM, Bloom PA, Cordeiro MF (2015) Advances in retinal ganglion cell imaging. Eye (Lond) 29:1260–1269. 10.1038/eye.2015.154 26293138 10.1038/eye.2015.154 PMC4815689

[11] Balusu S, De Strooper B (2024) The necroptosis cell death pathway drives neurodegeneration in Alzheimer’s disease. Acta Neuropathol 147:96. 10.1007/s00401-024-02747-5 38852117 10.1007/s00401-024-02747-5 PMC11162975

[12] Besser L, Kukull W, Knopman DS, Chui H, Galasko D, Weintraub S, Jicha G, Carlsson C, Burns J, Quinn J et al (2018) Version 3 of the national alzheimer’s coordinating center’s uniform data set. Alzheimer Dis Assoc Disord 32(4):351–358. 10.1097/WAD.0000000000000279 10.1097/WAD.0000000000000279 PMC6249084 30376508

[13] Bevan RJ, Hughes TR, Williams PA, Good MA, Morgan BP, Morgan JE (2020) Retinal ganglion cell degeneration correlates with hippocampal spine loss in experimental Alzheimer’s disease. Acta Neuropathol Commun 8(1):216. 10.1186/s40478-020-01094-2 33287900 10.1186/s40478-020-01094-2 PMC7720390

[14] Blair LJ, Nordhues BA, Hill SE, Scaglione KM, O’Leary JC 3rd, Fontaine SN, Breydo L, Zhang B, Li P, Wang L et al (2013) Accelerated neurodegeneration through chaperone-mediated oligomerization of tau. J Clin Invest 123(10):4158–4169. 10.1172/JCI69003 23999428 10.1172/JCI69003 PMC3784538

[15] Blanks JC, Hinton DR, Sadun AA, Miller CA (1989) Retinal ganglion cell degeneration in Alzheimer’s disease. Brain Res 501:364–372. 10.1016/0006-8993(89)90653-7 2819446 10.1016/0006-8993(89)90653-7

[16] Blanks JC, Schmidt SY, Torigoe Y, Porrello KV, Hinton DR, Blanks RH (1996) Retinal pathology in Alzheimer’s disease. II. Regional neuron loss and glial changes in GCL. Neurobiol Aging 17(3):385–395. 10.1016/0197-4580(96)00009-7 8725900 10.1016/0197-4580(96)00009-7

[17] Blanks JC, Torigoe Y, Hinton DR, Blanks RH (1996) Retinal pathology in Alzheimer’s disease. I. Ganglion cell loss in foveal/parafoveal retina. Neurobiol Aging 17(3):377–384. 10.1016/0197-4580(96)00010-3 8725899 10.1016/0197-4580(96)00010-3

[18] Bloom GS (2014) Amyloid-beta and tau: the trigger and bullet in Alzheimer disease pathogenesis. JAMA Neurol 71(4):505–508. 10.1001/jamaneurol.2013.5847 24493463 10.1001/jamaneurol.2013.5847 PMC12908160

[19] Braak H, Alafuzoff I, Arzberger T, Kretzschmar H, Del Tredici K (2006) Staging of Alzheimer disease-associated neurofibrillary pathology using paraffin sections and immunocytochemistry. Acta Neuropathol 112(4):389–404. 10.1007/s00401-006-0127-z 16906426 10.1007/s00401-006-0127-z PMC3906709

[20] Brier MR, Gordon B, Friedrichsen K, McCarthy J, Stern A, Christensen J, Owen C, Aldea P, Su Y, Hassenstab J et al (2016) Tau and Abeta imaging, CSF measures, and cognition in Alzheimer’s disease. Sci Transl Med 8:338ra66. 10.1126/scitranslmed.aaf2362 10.1126/scitranslmed.aaf2362 PMC5267531 27169802

[21] Caccamo A, Branca C, Piras IS, Ferreira E, Huentelman MJ, Liang WS, Readhead B, Dudley JT, Spangenberg EE, Green KN et al (2017) Necroptosis activation in Alzheimer’s disease. Nat Neurosci 20(9):1236–1246. 10.1038/nn.4608 10.1038/nn.4608 28758999

[22] Cao KJ, Kim JH, Kroeger H, Gaffney PM, Lin JH, Sigurdson CJ, Yang J (2021) ARCAM-1 facilitates fluorescence detection of amyloid-containing deposits in the Retina. Transl Vis Sci Technol 10(7):5. 10.1167/tvst.10.7.5 34096989 10.1167/tvst.10.7.5 PMC8185402

[23] Chen S, Zhang D, Zheng H, Cao T, Xia K, Su M, Meng Q (2023) The association between retina thinning and hippocampal atrophy in Alzheimer’s disease and mild cognitive impairment: a meta-analysis and systematic review. Front Aging Neurosci 15:1232941. 10.3389/fnagi.2023.1232941 37680540 10.3389/fnagi.2023.1232941 PMC10481874

[24] Chiasseu M, Alarcon-Martinez L, Belforte N, Quintero H, Dotigny F, Destroismaisons L, Vande Velde C, Panayi F, Louis C, Di Polo A (2017) Tau accumulation in the retina promotes early neuronal dysfunction and precedes brain pathology in a mouse model of Alzheimer’s disease. Mol Neurodegener 12(1):58. 10.1186/s13024-017-0199-3 28774322 10.1186/s13024-017-0199-3 PMC5543446

[25] Colom-Cadena M, Davies C, Sirisi S, Lee JE, Simzer EM, Tzioras M, Querol-Vilaseca M, Sanchez-Aced E, Chang YY, Holt K et al (2023) Synaptic oligomeric tau in Alzheimer’s disease - A potential culprit in the spread of tau pathology through the brain. Neuron 111(14):2170–2183.e6. 10.1016/j.neuron.2023.04.020 10.1016/j.neuron.2023.04.020 37192625

[26] Coppola G, Di Renzo A, Ziccardi L, Martelli F, Fadda A, Manni G, Barboni P, Pierelli F, Sadun AA, Parisi V (2015) Optical coherence tomography in Alzheimer’s Disease: a Meta-analysis. PLoS ONE 10(8):e0134750. 10.1371/journal.pone.0134750 26252902 10.1371/journal.pone.0134750 PMC4529274

[27] Cordeiro MF, Hill D, Patel R, Corazza P, Maddison J, Younis S (2022) Detecting retinal cell stress and apoptosis with DARC: progression from lab to clinic. Prog Retin Eye Res 86:100976. 10.1016/j.preteyeres.2021.100976 34102318 10.1016/j.preteyeres.2021.100976

[28] den Haan J, Morrema THJ, Verbraak FD, de Boer JF, Scheltens P, Rozemuller AJ, Bergen AAB, Bouwman FH, Hoozemans JJ (2018) Amyloid-beta and phosphorylated tau in post-mortem Alzheimer’s disease retinas. Acta Neuropathol Commun 6(1):147. 10.1186/s40478-018-0650-x 30593285 10.1186/s40478-018-0650-x PMC6309096

[29] Dong Y, Yu H, Li X, Bian K, Zheng Y, Dai M, Feng X, Sun Y, He Y, Yu B et al (2022) Hyperphosphorylated tau mediates neuronal death by inducing necroptosis and inflammation in Alzheimer’s disease. J Neuroinflammation 19:205. 10.1186/s12974-022-02567-y 35971179 10.1186/s12974-022-02567-y PMC9377071

[30] Du X, Koronyo Y, Mirzaei N, Yang C, Fuchs DT, Black KL, Koronyo-Hamaoui M, Gao L (2022) Label-free hyperspectral imaging and deep-learning prediction of retinal amyloid beta-protein and phosphorylated tau. PNAS Nexus 1(4):pgac164. 10.1093/pnasnexus/pgac164 36157597 10.1093/pnasnexus/pgac164 PMC9491695

[31] Dumitrascu OM, Lyden PD, Torbati T, Sheyn J, Sherzai A, Sherzai D, Sherman DS, Rosenberry R, Cheng S, Johnson KO et al (2020) Sectoral segmentation of retinal amyloid imaging in subjects with cognitive decline. Alzheimers Dement (Amst) 12(1):e12109. 10.1002/dad2.12109 10.1002/dad2.12109 PMC7521595 33015311

[32] Dumitrascu OM, Rosenberry R, Sherman DS, Khansari MM, Sheyn J, Torbati T, Sherzai A, Sherzai D, Johnson KO, Czeszynski AD et al (2021) Retinal Venular Tortuosity jointly with retinal amyloid Burden correlates with Verbal Memory loss: a pilot study. Cells 10(11):2926. 10.3390/cells10112926 10.3390/cells10112926 PMC8616417 34831149

[33] Elvira-Hurtado L, Lopez-Cuenca I, de Hoz R, Salas M, Sanchez-Puebla L, Ramirez-Torano F, Matamoros JA, Fernandez-Albarral JA, Rojas P Alfonsin S (2023) Alzheimer’s disease: a continuum with visual involvements. Front Psychol 14: 1124830 10.3389/fpsyg.2023.1124830 10.3389/fpsyg.2023.1124830 PMC10359162 37484098

[34] Erskine L, Herrera E (2014) Connecting the retina to the brain. ASN Neuro 6(6):1759091414562107. 10.1177/1759091414562107 10.1177/1759091414562107 PMC4720220 25504540

[35] Folstein MF, Folstein SE, McHugh PR (1975) Mini-mental state. A practical method for grading the cognitive state of patients for the clinician. J Psychiatr Res 12:189–198. 10.1016/0022-3956(75)90026-6 1202204 10.1016/0022-3956(75)90026-6

[36] Frisen L, Quigley HA (1984) Visual acuity in optic atrophy: a quantitative clinicopathological analysis. Graefes Arch Clin Exp Ophthalmol 222:71–74. 10.1007/BF02150634 6519442 10.1007/BF02150634

[37] Frontzkowski L, Ewers M, Brendel M, Biel D, Ossenkoppele R, Hager P, Steward A, Dewenter A, Romer S, Rubinski A et al (2022) Earlier Alzheimer’s disease onset is associated with tau pathology in brain hub regions and facilitated tau spreading. Nat Commun 13:4899. 10.1038/s41467-022-32592-7 35987901 10.1038/s41467-022-32592-7 PMC9392750

[38] Gaire BP, Koronyo Y, Fuchs D-T, Shi H, Rentsendorj A, Danziger R, Vit J-PS, Mirzaei N, Doustar J, Sheyn J et al (2024) Alzheimer’s disease pathophysiology in the retina. Prog Retinal Eye Res 101:101273. 10.1016/j.preteyeres.2024.101273 10.1016/j.preteyeres.2024.101273 PMC11285518 38759947

[39] Gastard MC, Troncoso JC, Koliatsos VE (2003) Caspase activation in the limbic cortex of subjects with early Alzheimer’s disease. Ann Neurol 54(3):393–398. 10.1002/ana.10680 12953274 10.1002/ana.10680

[40] Grimaldi A, Pediconi N, Oieni F, Pizzarelli R, Rosito M, Giubettini M, Santini T, Limatola C, Ruocco G, Ragozzino D et al (2019) Neuroinflammatory processes, A1 astrocyte activation and protein aggregation in the retina of Alzheimer’s disease patients, possible biomarkers for early diagnosis. Front Neurosci 13:925. 10.3389/fnins.2019.00925 31551688 10.3389/fnins.2019.00925 PMC6737046

[41] Groot C, Smith R, Stomrud E, Binette AP, Leuzy A, Wuestefeld A, Wisse LEM, Palmqvist S, Mattsson-Carlgren N Janelidze S (2023) phospho-tau with subthreshold tau-PET predicts increased tau accumulation rates in amyloid-positive individuals. Brain 146(4):1580–1591. 10.1093/brain/awac329 10.1093/brain/awac329 PMC10115173 36084009

[42] Grundke-Iqbal I, Iqbal K, Tung YC, Quinlan M, Wisniewski HM, Binder LI (1986) Abnormal phosphorylation of the microtubule-associated protein tau (tau) in Alzheimer cytoskeletal pathology. Proc Natl Acad Sci U S A 83:4913–4917. 10.1073/pnas.83.13.4913 3088567 10.1073/pnas.83.13.4913 PMC323854

[43] Hadoux X, Hui F, Lim JKH, Masters CL, Pebay A, Chevalier S, Ha J, Loi S, Fowler CJ, Rowe C et al (2019) Non-invasive in vivo hyperspectral imaging of the retina for potential biomarker use in Alzheimer’s disease. Nat Commun 10:4227. 10.1038/s41467-019-12242-1 31530809 10.1038/s41467-019-12242-1 PMC6748929

[44] Hammer DX, Agrawal A, Villanueva R, Saeedi O, Liu Z (2020) Label-free adaptive optics imaging of human retinal macrophage distribution and dynamics. Proc Natl Acad Sci USA 117(48):30661–30669. 10.1073/pnas.2010943117 33168747 10.1073/pnas.2010943117 PMC7720180

[45] Hart de Ruyter FJ, Evers M, Morrema THJ, Dijkstra AA, den Haan J, Twisk JWR, de Boer JF, Scheltens P, Bouwman FH, Verbraak FD et al (2024) Neuropathological hallmarks in the post-mortem retina of neurodegenerative diseases. Acta Neuropathol 148(1):24. 10.1007/s00401-024-02769-z 39160362 10.1007/s00401-024-02769-z PMC11333524

[46] Hart de Ruyter FJ, Morrema THJ, den Haan J, Netherlands Brain B, Twisk JWR, de Boer JF, Scheltens P, Boon BDC, Thal DR, Rozemuller AJ et al (2022) Phosphorylated tau in the retina correlates with tau pathology in the brain in Alzheimer’s disease and primary tauopathies. Acta Neuropathol 145(2):197–218. 10.1007/s00401-022-02525-1 36480077 10.1007/s00401-022-02525-1

[47] Hart NJ, Koronyo Y, Black KL, Koronyo-Hamaoui M (2016) Ocular indicators of Alzheimer’s: exploring disease in the retina. Acta Neuropathol 132(6):767–787. 10.1007/s00401-016-1613-6 27645291 10.1007/s00401-016-1613-6 PMC5106496

[48] Hinton DR, Sadun AA, Blanks JC, Miller CA (1986) Optic-nerve degeneration in Alzheimer’s disease. N Engl J Med 315:485–487. 10.1056/NEJM198608213150804 3736630 10.1056/NEJM198608213150804

[49] Hoozemans JJ, van Haastert ES, Nijholt DA, Rozemuller AJ, Eikelenboom P, Scheper W (2009) The unfolded protein response is activated in pretangle neurons in Alzheimer’s disease hippocampus. Am J Pathol 174:1241–1251. 10.2353/ajpath.2009.080814 19264902 10.2353/ajpath.2009.080814 PMC2671357

[50] Hyman BT, Phelps CH, Beach TG, Bigio EH, Cairns NJ, Carrillo MC, Dickson DW, Duyckaerts C, Frosch MP, Masliah E et al (2012) National Institute on Aging-Alzheimer’s Association guidelines for the neuropathologic assessment of Alzheimer’s disease. Alzheimers Dement 8:1–13. 10.1016/j.jalz.2011.10.007 22265587 10.1016/j.jalz.2011.10.007 PMC3266529

[51] Iqbal K, Wisniewski HM, Shelanski ML, Brostoff S, Liwnicz BH, Terry RD (1974) Protein changes in senile dementia. Brain Res 77:337–343. 10.1016/0006-8993(74)90798-7 4604978 10.1016/0006-8993(74)90798-7

[52] Jack CR Jr., Bennett DA, Blennow K, Carrillo MC, Dunn B, Haeberlein SB, Holtzman DM, Jagust W, Jessen F, Karlawish J et al (2018) NIA-AA Research Framework: Toward a biological definition of Alzheimer’s disease. Alzheimers Dement 14:535–562 10.1016/j.jalz.2018.02.018 10.1016/j.jalz.2018.02.018 PMC5958625 29653606

[53] Kadar A, Wittmann G, Liposits Z, Fekete C (2009) Improved method for combination of immunocytochemistry and nissl staining. J Neurosci Methods 184:115–118. 10.1016/j.jneumeth.2009.07.010 19615409 10.1016/j.jneumeth.2009.07.010 PMC2753838

[54] Katz B, Rimmer S, Iragui V, Katzman R (1989) Abnormal pattern electroretinogram in Alzheimer’s disease: evidence for retinal ganglion cell degeneration? Ann Neurol 26:221–225. 10.1002/ana.410260207 2774509 10.1002/ana.410260207

[55] Kfoury N, Holmes BB, Jiang H, Holtzman DM, Diamond MI (2012) Trans-cellular propagation of tau aggregation by fibrillar species. J Biol Chem 287:19440–19451. 10.1074/jbc.M112.346072 22461630 10.1074/jbc.M112.346072 PMC3365982

[56] Kim US, Mahroo OA, Mollon JD, Yu-Wai-Man P (2021) Retinal ganglion cells-diversity of cell types and clinical relevance. Front Neurol 12:661938. 10.3389/fneur.2021.661938 34093409 10.3389/fneur.2021.661938 PMC8175861

[57] Kirbas S, Turkyilmaz K, Anlar O, Tufekci A, Durmus M (2013) Retinal nerve fiber layer thickness in patients with Alzheimer disease. J Neuroophthalmol 33:58–61. 10.1097/WNO.0b013e318267fd5f 22918296 10.1097/WNO.0b013e318267fd5f

[58] Kivela T (1998) Parvalbumin, a horizontal cell-associated calcium-binding protein in retinoblastoma eyes. Invest Ophthalmol Vis Sci 39:1044–1048 9579485

[59] Kopeikina KJ, Hyman BT, Spires-Jones TL (2012) Soluble forms of tau are toxic in Alzheimer’s disease. Transl Neurosci 3:223–233. 10.2478/s13380-012-0032-y 23029602 10.2478/s13380-012-0032-y PMC3460520

[60] Koper MJ, Moonen S, Ronisz A, Ospitalieri S, Callaerts-Vegh Z, T’Syen D, Rabe S, Staufenbiel M, De Strooper B, Balusu S et al (2024) Inhibition of an Alzheimer’s disease-associated form of necroptosis rescues neuronal death in mouse models. Sci Transl Med 16:eadf5128. 10.1126/scitranslmed.adf5128 39475569 10.1126/scitranslmed.adf5128

[61] Koper MJ, Tome SO, Gawor K, Belet A, Van Schoor E, Schaeverbeke J, Vandenberghe R, Vandenbulcke M, Ghebremedhin E Otto M (2022) LATE-NC aggravates GVD-mediated necroptosis in Alzheimer’s disease. Acta Neuropathol Commun 10:128. 10.1186/s40478-022-01432-6 10.1186/s40478-022-01432-6 PMC9441100 36057624

[62] Koper MJ, Van Schoor E, Ospitalieri S, Vandenberghe R, Vandenbulcke M, von Arnim CAF, Tousseyn T, Balusu S, De Strooper B, Thal DR (2020) Necrosome complex detected in granulovacuolar degeneration is associated with neuronal loss in Alzheimer’s disease. Acta Neuropathol 139:463–484. 10.1007/s00401-019-02103-y 31802237 10.1007/s00401-019-02103-y

[63] Koronyo Y, Biggs D, Barron E, Boyer DS, Pearlman JA, Au WJ, Kile SJ, Blanco A, Fuchs DT, Ashfaq A et al (2017) Retinal amyloid pathology and proof-of-concept imaging trial in Alzheimer’s disease. JCI Insight 2(16):e93621. 10.1172/jci.insight.93621 10.1172/jci.insight.93621 PMC5621887 28814675

[64] Koronyo Y, Rentsendorj A, Mirzaei N, Regis GC, Sheyn J, Shi H, Barron E, Cook-Wiens G, Rodriguez AR, Medeiros R et al (2023) Retinal pathological features and proteome signatures of Alzheimer’s disease. Acta Neuropathol 145(4):409–438. 10.1007/s00401-023-02548-2 36773106 10.1007/s00401-023-02548-2 PMC10020290

[65] Koronyo-Hamaoui M, Koronyo Y, Ljubimov AV, Miller CA, Ko MK, Black KL, Schwartz M, Farkas DL (2011) Identification of amyloid plaques in retinas from Alzheimer’s patients and noninvasive in vivo optical imaging of retinal plaques in a mouse model. NeuroImage 54(Suppl 1):S204–217. 10.1016/j.neuroimage.2010.06.020 20550967 10.1016/j.neuroimage.2010.06.020 PMC2991559

[66] Kwong JM, Caprioli J, Piri N (2010) RNA binding protein with multiple splicing: a new marker for retinal ganglion cells. Invest Ophthalmol Vis Sci 51:1052–1058. 10.1167/iovs.09-4098 19737887 10.1167/iovs.09-4098 PMC3979483

[67] Kyalu Ngoie Zola N, Balty C, Pyr Dit Ruys S, Vanparys AAT, Huyghe NDG, Herinckx G, Johanns M, Boyer E, Kienlen-Campard P, Rider MH al (2023) Specific post-translational modifications of soluble tau protein distinguishes Alzheimer’s disease and primary tauopathies. Nat Commun 14:3706. 10.1038/s41467-023-39328-1 37349319 10.1038/s41467-023-39328-1 PMC10287718

[68] La Joie R, Visani AV, Baker SL, Brown JA, Bourakova V, Cha J, Chaudhary K, Edwards L, Iaccarino L, Janabi M et al (2020) Prospective longitudinal atrophy in Alzheimer’s disease correlates with the intensity and topography of baseline tau-PET. Sci Transl Med 12(524):eaau5732. 10.1126/scitranslmed.aau5732 10.1126/scitranslmed.aau5732 PMC7035952 31894103

[69] La Morgia C, Di Vito L, Carelli V, Carbonelli M (2017) Patterns of retinal ganglion cell damage in neurodegenerative disorders: parvocellular vs Magnocellular Degeneration in Optical Coherence Tomography studies. Front Neurol 8:710. 10.3389/fneur.2017.00710 29312131 10.3389/fneur.2017.00710 PMC5744067

[70] La Morgia C, Ross-Cisneros FN, Koronyo Y, Hannibal J, Gallassi R, Cantalupo G, Sambati L, Pan BX, Tozer KR, Barboni P et al (2016) Melanopsin retinal ganglion cell loss in Alzheimer disease. Ann Neurol 79:90–109. 10.1002/ana.24548 26505992 10.1002/ana.24548 PMC4737313

[71] Lambert MP, Barlow AK, Chromy BA, Edwards C, Freed R, Liosatos M, Morgan TE, Rozovsky I, Trommer B, Viola KL al (1998) Diffusible, nonfibrillar ligands derived from Abeta1-42 are potent central nervous system neurotoxins. Proc Natl Acad Sci U S A 95:6448–6453. 10.1073/pnas.95.11.6448 9600986 10.1073/pnas.95.11.6448 PMC27787

[72] Laycock R, Crewther SG, Crewther DP (2007) A role for the ‘magnocellular advantage’ in visual impairments in neurodevelopmental and psychiatric disorders. Neurosci Biobehav Rev 31:363–376. 10.1016/j.neubiorev.2006.10.003 17141311 10.1016/j.neubiorev.2006.10.003

[73] Lee S, Jiang K, McIlmoyle B, To E, Xu QA, Hirsch-Reinshagen V, Mackenzie IR, Hsiung GR, Eadie BD, Sarunic MV et al (2020) Amyloid Beta immunoreactivity in the retinal ganglion cell layer of the Alzheimer’s Eye. Front Neurosci 14:758. 10.3389/fnins.2020.00758 32848548 10.3389/fnins.2020.00758 PMC7412634

[74] Lemmens S, Van Craenendonck T, Van Eijgen J, De Groef L, Bruffaerts R, de Jesus DA, Charle W, Jayapala M, Sunaric-Megevand G, Standaert A et al (2020) Combination of snapshot hyperspectral retinal imaging and optical coherence tomography to identify Alzheimer’s disease patients. Alzheimers Res Ther 12:144. 10.1186/s13195-020-00715-1 33172499 10.1186/s13195-020-00715-1 PMC7654576

[75] Lopez-Cuenca I, Nebreda A, Garcia-Colomo A, Salobrar-Garcia E, de Frutos-Lucas J, Bruna R, Ramirez AI, Ramirez-Torano F, Salazar JJ, Barabash A et al (2023) Early visual alterations in individuals at-risk of Alzheimer’s disease: a multidisciplinary approach. Alzheimers Res Ther 15:19. 10.1186/s13195-023-01166-0 36694201 10.1186/s13195-023-01166-0 PMC9872347

[76] Lopez-de-Eguileta A, Lopez-Garcia S, Lage C, Pozueta A, Garcia-Martinez M, Kazimierczak M, Bravo M, Irure J, Lopez-Hoyos M, Munoz-Cacho P et al (2022) The retinal ganglion cell layer reflects neurodegenerative changes in cognitively unimpaired individuals. Alzheimers Res Ther 14:57. 10.1186/s13195-022-00998-6 35449033 10.1186/s13195-022-00998-6 PMC9022357

[77] Lu Y, Li Z, Zhang X, Ming B, Jia J, Wang R, Ma D (2010) Retinal nerve fiber layer structure abnormalities in early Alzheimer’s disease: evidence in optical coherence tomography. Neurosci Lett 480:69–72. 10.1016/j.neulet.2010.06.006 20609426 10.1016/j.neulet.2010.06.006

[78] Marziani E, Pomati S, Ramolfo P, Cigada M, Giani A, Mariani C, Staurenghi G (2013) Evaluation of retinal nerve fiber layer and ganglion cell layer thickness in Alzheimer’s disease using spectral-domain optical coherence tomography. Invest Ophthalmol Vis Sci 54:5953–5958. 10.1167/iovs.13-12046 23920375 10.1167/iovs.13-12046

[79] Miller DT, Kurokawa K (2020) Cellular-Scale imaging of transparent retinal structures and processes using adaptive Optics Optical Coherence Tomography. Annu Rev Vis Sci 6:115–148. 10.1146/annurev-vision-030320-041255 32609578 10.1146/annurev-vision-030320-041255 PMC7864592

[80] Mirra SS, Heyman A, McKeel D, Sumi SM, Crain BJ, Brownlee LM, Vogel FS, Hughes JP, van Belle G, Berg L (1991) The Consortium to establish a Registry for Alzheimer’s Disease (CERAD). Part II. Standardization of the neuropathologic assessment of Alzheimer’s disease. Neurology 41:479–486. 10.1212/wnl.41.4.479 2011243 10.1212/wnl.41.4.479

[81] Mirzaei N, Shi H, Oviatt M, Doustar J, Rentsendorj A, Fuchs DT, Sheyn J, Black KL, Koronyo Y, Koronyo-Hamaoui M (2020) Alzheimer’s retinopathy: seeing Disease in the eyes. Front Neurosci 14:921. 10.3389/fnins.2020.00921 33041751 10.3389/fnins.2020.00921 PMC7523471

[82] Moloney CM, Lowe VJ, Murray ME (2021) Visualization of neurofibrillary tangle maturity in Alzheimer’s disease: a clinicopathologic perspective for biomarker research. Alzheimers Dement 17:1554–1574. 10.1002/alz.12321 33797838 10.1002/alz.12321 PMC8478697

[83] Montine TJ, Phelps CH, Beach TG, Bigio EH, Cairns NJ, Dickson DW, Duyckaerts C, Frosch MP, Masliah E, Mirra SS et al (2012) National Institute on Aging-Alzheimer’s Association guidelines for the neuropathologic assessment of Alzheimer’s disease: a practical approach. Acta Neuropathol 123:1–11. 10.1007/s00401-011-0910-3 22101365 10.1007/s00401-011-0910-3 PMC3268003

[84] More SS, Beach JM, McClelland C, Mokhtarzadeh A, Vince R (2019) In vivo Assessment of retinal biomarkers by Hyperspectral Imaging: early detection of Alzheimer’s Disease. ACS Chem Neurosci 10:4492–4501. 10.1021/acschemneuro.9b00331 31603648 10.1021/acschemneuro.9b00331

[85] Morris JC (1993) The clinical dementia rating (CDR): current version and scoring rules. Neurology 43:2412–2414. 10.1212/wnl.43.11.2412-a 8232972 10.1212/wnl.43.11.2412-a

[86] Mucke L, Selkoe DJ (2012) Neurotoxicity of amyloid beta-protein: synaptic and network dysfunction. Cold Spring Harb Perspect Med 2:a006338. 10.1101/cshperspect.a006338 22762015 10.1101/cshperspect.a006338 PMC3385944

[87] Nadal-Nicolás FM, Galindo-Romero C, Lucas-Ruiz F, Marsh-Amstrong N, Li W, Vidal-Sanz M, Agudo-Barriuso M (2023) Pan-retinal ganglion cell markers in mice, rats, and rhesus macaques. Zool Res 44:226–248. 10.24272/j.issn.2095-8137.2022.308 36594396 10.24272/j.issn.2095-8137.2022.308 PMC9841181

[88] Ngolab J, Donohue M, Belsha A, Salazar J, Cohen P, Jaiswal S, Tan V, Gessert D, Korouri S, Aggarwal NT et al (2021) Feasibility study for detection of retinal amyloid in clinical trials: The Anti-Amyloid Treatment in Asymptomatic Alzheimer’s Disease (A4) trial. Alzheimers Dement (Amst) 13:e12199. 10.1002/dad2.12199 10.1002/dad2.12199 PMC8369843 34430703

[89] Nunez-Diaz C, Andersson E, Schultz N, Poceviciute D, Hansson O, Netherlands Brain B, Nilsson KPR, Wennstrom M (2024) The fluorescent ligand bTVBT2 reveals increased p-tau uptake by retinal microglia in Alzheimer’s disease patients and App(NL-F/NL-F) mice. Alzheimers Res Ther 16(1):4. 10.1186/s13195-023-01375-7 10.1186/s13195-023-01375-7 PMC10763304 38167557

[90] O’Bryant SE, Humphreys JD, Smith GE, Ivnik RJ, Graff-Radford NR, Petersen RC, Lucas JA (2008) Detecting dementia with the mini-mental state examination in highly educated individuals. Arch Neurol 65:963–967. 10.1001/archneur.65.7.963 18625866 10.1001/archneur.65.7.963 PMC2587038

[91] Oku H, Kida T, Horie T, Taki K, Mimura M, Kojima S, Ikeda T (2019) Tau is involved in death of retinal ganglion cells of rats from Optic nerve crush. Invest Ophthalmol Vis Sci 60:2380–2387. 10.1167/iovs.19-26683 31141609 10.1167/iovs.19-26683

[92] Pasparakis M, Vandenabeele P (2015) Necroptosis and its role in inflammation. Nature 517:311–320. 10.1038/nature14191 25592536 10.1038/nature14191

[93] Peeraer E, Bottelbergs A, Van Kolen K, Stancu IC, Vasconcelos B, Mahieu M, Duytschaever H, Ver Donck L, Torremans A, Sluydts E et al (2015) Intracerebral injection of preformed synthetic tau fibrils initiates widespread tauopathy and neuronal loss in the brains of tau transgenic mice. Neurobiol Dis 73:83–95. 10.1016/j.nbd.2014.08.032 25220759 10.1016/j.nbd.2014.08.032 PMC4303592

[94] Pereiro X, Ruzafa N, Urcola JH, Sharma SC, Vecino E (2020) Differential distribution of RBPMS in Pig, Rat, and human retina after Damage. Int J Mol Sci 21(23):9330. 10.3390/ijms21239330 10.3390/ijms21239330 PMC7729751 33297577

[95] Piri N, Kwong JM, Song M, Caprioli J (2006) Expression of hermes gene is restricted to the ganglion cells in the retina. Neurosci Lett 405:40–45. 10.1016/j.neulet.2006.06.049 16870336 10.1016/j.neulet.2006.06.049

[96] Puangmalai N, Bhatt N, Montalbano M, Sengupta U, Gaikwad S, Ventura F, McAllen S, Ellsworth A, Garcia S, Kayed R (2020) Internalization mechanisms of brain-derived tau oligomers from patients with Alzheimer’s disease, progressive supranuclear palsy and dementia with Lewy bodies. Cell Death Dis 11:314. 10.1038/s41419-020-2503-3 32366836 10.1038/s41419-020-2503-3 PMC7198578

[97] Purves DAGJ, Fitzpatrick D, Katz LC, LaMantia A-S, McNamara JO, William SM (2001) Neuroscience, 2nd edition. Sunderland (MA): Sinauer Associates, City

[98] Qiu Y, Jin T, Mason E, Campbell MCW (2020) Predicting Thioflavin fluorescence of retinal amyloid deposits Associated with Alzheimer’s Disease from their Polarimetric Properties. Transl Vis Sci Technol 9:47. 10.1167/tvst.9.2.47 32879757 10.1167/tvst.9.2.47 PMC7443113

[99] Rawat P, Sehar U, Bisht J, Selman A, Culberson J, Reddy PH (2022) Phosphorylated Tau in Alzheimer’s Disease and Other Tauopathies. Int J Mol Sci 23(21):12841. 10.3390/ijms232112841 10.3390/ijms232112841 PMC9654278 36361631

[100] Regalado-Reyes M, Furcila D, Hernandez F, Avila J, DeFelipe J, Leon-Espinosa G (2019) Phospho-tau changes in the human CA1 during Alzheimer’s Disease Progression. J Alzheimers Dis 69:277–288. 10.3233/JAD-181263 30958368 10.3233/JAD-181263 PMC6598029

[101] Risacher SL, WuDunn D, Tallman EF, West JD, Gao S, Farlow MR, Brosch JR, Apostolova LG, Saykin AJ (2020) Visual contrast sensitivity is associated with the presence of cerebral amyloid and tau deposition. Brain Commun 2:fcaa019. 10.1093/braincomms/fcaa019 32309804 10.1093/braincomms/fcaa019 PMC7151662

[102] Rodriguez AR, de Sevilla Müller LP, Brecha NC (2014) The RNA binding protein RBPMS is a selective marker of ganglion cells in the mammalian retina. J Comp Neurol 522:1411–1443. 10.1002/cne.23521 24318667 10.1002/cne.23521 PMC3959221

[103] Rossetti HC, Munro Cullum C, Hynan LS, Lacritz LH (2010) The CERAD neuropsychologic battery total score and the progression of Alzheimer disease. Alzheimer Dis Assoc Disord 24:138–142. 10.1097/WAD.0b013e3181b76415 20505431 10.1097/WAD.0b013e3181b76415 PMC2920638

[104] Sadun AA, Borchert M, DeVita E, Hinton DR, Bassi CJ (1987) Assessment of visual impairment in patients with Alzheimer’s disease. Am J Ophthalmol 104:113–120. 10.1016/0002-9394(87)90001-8 3618708 10.1016/0002-9394(87)90001-8

[105] Salobrar-Garcia E, de Hoz R, Ramirez AI, Lopez-Cuenca I, Rojas P, Vazirani R, Amarante C, Yubero R, Gil P, Pinazo-Duran MD et al (2019) Changes in visual function and retinal structure in the progression of Alzheimer’s disease. PLoS ONE 14:e0220535. 10.1371/journal.pone.0220535 31415594 10.1371/journal.pone.0220535 PMC6695171

[106] Sanes JR, Masland RH (2015) The types of retinal ganglion cells: current status and implications for neuronal classification. Annu Rev Neurosci 38:221–246. 10.1146/annurev-neuro-071714-034120 25897874 10.1146/annurev-neuro-071714-034120

[107] Schön C, Hoffmann NA, Ochs SM, Burgold S, Filser S, Steinbach S, Seeliger MW, Arzberger T, Goedert M, Kretzschmar HA al (2012) Long-term in vivo imaging of fibrillar tau in the retina of P301S transgenic mice. PLoS ONE 7:e53547. 10.1371/journal.pone.0053547 23300938 10.1371/journal.pone.0053547 PMC3534024

[108] Schultz N, Byman E, Wennström M (2020) Levels of retinal Amyloid-β correlate with levels of retinal IAPP and hippocampal Amyloid-β in Neuropathologically evaluated individuals. J Alzheimers Dis 73:1201–1209. 10.3233/jad-190868 31884473 10.3233/JAD-190868 PMC7081096

[109] Serrano-Pozo A, Frosch MP, Masliah E, Hyman BT (2011) Neuropathological alterations in Alzheimer disease. Cold Spring Harb Perspect Med 1:a006189. 10.1101/cshperspect.a006189 22229116 10.1101/cshperspect.a006189 PMC3234452

[110] Shi H, Koronyo Y, Fuchs DT, Sheyn J, Jallow O, Mandalia K, Graham SL, Gupta VK, Mirzaei M, Kramerov AA et al (2023) Retinal arterial Aβ_40_ deposition is linked with tight junction loss and cerebral amyloid angiopathy in MCI and AD patients. Alzheimers Dement 19(11):5185–5197. 10.1002/alz.13086 10.1002/alz.13086 PMC10638467 37166032

[111] Shi H, Koronyo Y, Rentsendorj A, Regis GC, Sheyn J, Fuchs DT, Kramerov AA, Ljubimov AV, Dumitrascu OM, Rodriguez AR (2020) Identification of early pericyte loss and vascular amyloidosis in Alzheimer’s disease retina. Acta Neuropathol 139(5):813–836. 10.1007/s00401-020-02134-w 10.1007/s00401-020-02134-w PMC7181564 32043162

[112] Shi H, Mirzaei N, Koronyo Y, Davis MR, Robinson E, Braun GM, Jallow O, Rentsendorj A, Ramanujan VK, Fert-Bober J et al (2024) Identification of retinal oligomeric, citrullinated, and other tau isoforms in early and advanced AD and relations to disease status. Acta Neuropathol 148(1):3. 10.1007/s00401-024-02760-8 10.1007/s00401-024-02760-8 PMC11233395 38980423

[113] Snyder PJ, Alber J, Alt C, Bain LJ, Bouma BE, Bouwman FH, DeBuc DC, Campbell MCW, Carrillo MC Chew EY (2021) Retinal imaging in Alzheimer’s and neurodegenerative diseases. Alzheimers Dement 17: 103–111 10.1002/alz.12179 10.1002/alz.12179 PMC8062064 33090722

[114] Sperling RA, Aisen PS, Beckett LA, Bennett DA, Craft S, Fagan AM, Iwatsubo T, Jack CR Jr., Kaye J, Montine, TJ et al (2011) Toward defining the preclinical stages of Alzheimer’s disease: recommendations from the National Institute on Aging-Alzheimer’s Association workgroups on diagnostic guidelines for Alzheimer’s disease. Alzheimers Dement 7:280–292. 10.1016/j.jalz.2011.03.003 10.1016/j.jalz.2011.03.003 PMC3220946 21514248

[115] Sperling RA, Donohue MC, Raman R, Sun CK, Yaari R, Holdridge K, Siemers E, Johnson KA, Aisen PS, Team AS (2020) Association of factors with elevated amyloid Burden in clinically normal older individuals. JAMA Neurol 77:735–745. 10.1001/jamaneurol.2020.0387 32250387 10.1001/jamaneurol.2020.0387 PMC7136861

[116] Su JH, Zhao M, Anderson AJ, Srinivasan A, Cotman CW (2001) Activated caspase-3 expression in Alzheimer’s and aged control brain: correlation with Alzheimer pathology. Brain Res 898:350–357. 10.1016/s0006-8993(01)02018-2 11306022 10.1016/s0006-8993(01)02018-2

[117] Taddei RN, Perbet R, Mate de Gerando A, Wiedmer AE, Sanchez-Mico M, Connors Stewart T, Gaona A, Melloni A, Amaral AC, Duff K et al (2023) Tau oligomer-containing synapse elimination by microglia and astrocytes in Alzheimer disease. JAMA Neurol 80:1209–1221. 10.1001/jamaneurol.2023.3530 37812432 10.1001/jamaneurol.2023.3530 PMC10562992

[118] Tadokoro K, Yamashita T, Kimura S, Nomura E, Ohta Y, Omote Y, Takemoto M, Hishikawa N, Morihara R, Morizane Y et al (2021) Retinal Amyloid Imaging for Screening Alzheimer’s Disease. J Alzheimers Dis 83:927–934 10.3233/JAD-210327 10.3233/JAD-210327 34366344

[119] Tai HC, Serrano-Pozo A, Hashimoto T, Frosch MP, Spires-Jones TL, Hyman BT (2012) The synaptic accumulation of hyperphosphorylated tau oligomers in Alzheimer disease is associated with dysfunction of the ubiquitin-proteasome system. Am J Pathol 181:1426–1435. 10.1016/j.ajpath.2012.06.033 22867711 10.1016/j.ajpath.2012.06.033 PMC3463637

[120] Tenhula WN, Xu SZ, Madigan MC, Heller K, Freeman WR, Sadun AA (1992) Morphometric comparisons of optic nerve axon loss in acquired immunodeficiency syndrome. Am J Ophthalmol 113:14–20. 10.1016/s0002-9394(14)75746-0 1370189 10.1016/s0002-9394(14)75746-0

[121] Thal DR, Gawor K, Moonen S (2024) Regulated cell death and its role in Alzheimer’s disease and amyotrophic lateral sclerosis. Acta Neuropathol 147:69. 10.1007/s00401-024-02722-0 38583129 10.1007/s00401-024-02722-0

[122] Thal DR, Rüb U, Orantes M, Braak H (2002) Phases of a beta-deposition in the human brain and its relevance for the development of AD. Neurology 58:1791–1800. 10.1212/wnl.58.12.1791 12084879 10.1212/wnl.58.12.1791

[123] Theofilas P, Ehrenberg AJ, Nguy A, Thackrey JM, Dunlop S, Mejia MB, Alho AT, Paraizo Leite RE, Rodriguez RD, Suemoto CK et al (2018) Probing the correlation of neuronal loss, neurofibrillary tangles, and cell death markers across the Alzheimer’s disease Braak stages: a quantitative study in humans. Neurobiol Aging 61:1–12 10.1016/j.neurobiolaging.2017.09.007 10.1016/j.neurobiolaging.2017.09.007 PMC5705284 29031088

[124] Thomsen ND, Koerber JT, Wells JA (2013) Structural snapshots reveal distinct mechanisms of procaspase-3 and– 7 activation. Proc Natl Acad Sci U S A 110:8477–8482. 10.1073/pnas.1306759110 23650375 10.1073/pnas.1306759110 PMC3666719

[125] Trick GL, Barris MC, Bickler-Bluth M (1989) Abnormal pattern electroretinograms in patients with senile dementia of the Alzheimer type. Ann Neurol 26:226–231. 10.1002/ana.410260208 2774510 10.1002/ana.410260208

[126] Uchihara T (2007) Silver diagnosis in neuropathology: principles, practice and revised interpretation. Acta Neuropathol 113:483–499. 10.1007/s00401-007-0200-2 17401570 10.1007/s00401-007-0200-2 PMC1868652

[127] van der Gaag BL, Deshayes NAC, Breve JJP, Bol J, Jonker AJ, Hoozemans JJM, Courade JP, van de Berg WDJ (2024) Distinct tau and alpha-synuclein molecular signatures in Alzheimer’s disease with and without Lewy bodies and Parkinson’s disease with dementia. Acta Neuropathol 147:14. 10.1007/s00401-023-02657-y 38198008 10.1007/s00401-023-02657-y PMC10781859

[128] VanderWall KB, Lu B, Alfaro JS, Allsop AR, Carr AS, Wang S, Meyer JS (2020) Differential susceptibility of retinal ganglion cell subtypes in acute and chronic models of injury and disease. Sci Rep 10:17359. 10.1038/s41598-020-71460-6 33060618 10.1038/s41598-020-71460-6 PMC7566630

[129] Walkiewicz G, Ronisz A, Van Ginderdeuren R, Lemmens S, Bouwman FH, Hoozemans JJM, Morrema THJ, Rozemuller AJ, Hart de Ruyter FJ, De Groef L et al (2024) Primary retinal tauopathy: a tauopathy with a distinct molecular pattern. Alzheimers Dement 20(1):330–340. 10.1002/alz.13424 10.1002/alz.13424 PMC10916964 37615275

[130] Wiersma VI, Hoozemans JJM, Scheper W (2020) Untangling the origin and function of granulovacuolar degeneration bodies in neurodegenerative proteinopathies. Acta Neuropathol Commun 8(1):153. 10.1186/s40478-020-00996-5 32883341 10.1186/s40478-020-00996-5 PMC7469111

[131] Xu QA, Boerkoel P, Hirsch-Reinshagen V, Mackenzie IR, Hsiung GR, Charm G, To EF, Liu AQ, Schwab K, Jiang K et al (2022) Muller cell degeneration and microglial dysfunction in the Alzheimer’s retina. Acta Neuropathol Commun 10:145. 10.1186/s40478-022-01448-y 36199154 10.1186/s40478-022-01448-y PMC9533552

[132] Yarns BC, Holiday KA, Carlson DM, Cosgrove CK, Melrose RJ (2022) Pathophysiology of Alzheimer’s Disease. Psychiatr Clin North Am 45:663–676. 10.1016/j.psc.2022.07.003 36396271 10.1016/j.psc.2022.07.003

[133] Zhang R, Song Y, Su X (2023) Necroptosis and Alzheimer’s Disease: pathogenic mechanisms and Therapeutic opportunities. J Alzheimers Dis 94:S367–S386. 10.3233/JAD-220809 36463451 10.3233/JAD-220809 PMC10473100

[134] Zhang Y, Chen H, Li R, Sterling K, Song W (2023) Amyloid beta-based therapy for Alzheimer’s disease: challenges, successes and future. Signal Transduct Target Ther 8:248. 10.1038/s41392-023-01484-7 37386015 10.1038/s41392-023-01484-7 PMC10310781

[135] Zhao B, Li Y, Fan Z, Wu Z, Shu J, Yang X, Yang Y, Wang X, Li B, Wang X et al (2024) Eye-brain connections revealed by multimodal retinal and brain imaging genetics. Nat Commun 15:6064. 10.1038/s41467-024-50309-w 39025851 10.1038/s41467-024-50309-w PMC11258354

