# Supplementary material for: Retinal ganglion cell vulnerability to pathogenic tau in Alzheimer’s disease
Source: Acta Neuropathol Commun. 2025 Feb 15;13:31. doi: 10.1186/s40478-025-01935-y (PMC11829413; doi:10.1186/s40478-025-01935-y)
Supplement: Supplementary file 1 — Supplementary Material 1 [file 40478_2025_1935_MOESM1_ESM.pdf]

Supplementary Online Content

Retinal ganglion cell vulnerability to pathogenic tau in Alzheimer's disease

Miyah R. Davis, Edward Robinson, Yosef Koronyo, Elena Salobrar-Garcia, Altan Rentsendorj, Bhakta P. Gaire, Nazanin Mirzaei, Rakez Kayed, Alfredo A. Sadun, Alexander V. Ljubimov, Lon S. Schneider, Debra Hawes, Keith L. Black, Dieu-Trang Fuchs, Maya Koronyo-Hamaoui[@]

[@]Corresponding author: Maya Koronyo-Hamaoui, PhD, Cedars-Sinai Medical Center, 127 S. San Vicente Blvd., A6212, Los Angeles, CA, USA 90048. Tel: (310)-423-7473, E-mail: maya.koronyo@csmc.edu

Supplementary Table 1. List of human donors in this study.

Supplementary Table 2. List of antibodies.

Supplementary Figure 1. Extended data on ganglion cell integrity in retinal tissues from MCI and AD patients.

Supplementary Figure 2. Extended data on phosphorylated tau in RGCs of MCI and AD patients.

Supplementary Figure 3. Extended data on oligomeric tau isoforms within RGCs of MCI and AD patients.

Supplementary Table 3. Correlations of pS396-tau or Oligo-tau RGCs with brain pathology and cognition in AD patients only.

Supplementary Table 1. List of human donors in this study.

Diagnosis	Sex	Race	Age at death	Thal A	Braak B	CERAD C	CAA Score	Co-morbid. [LB/AS VD]	Braak Stage	CDR Score	MMSE Score	APOE status
AD1	M	W	79	3	3	3	1.5	-/+	V	n.a.	n.a.	n.a.
AD2	M	H	97	3	2	3	1	-/+	III	1	26	e3/e3
AD3	M	W	90	3	3	3	2	-/+	VI	3	n.a.	n.a.
AD4	M	W	90	3	2	3	1	-/+	IV	3	n.a.	e3/e4
AD5	F	W	90	3	3	3	1	-/+	V-VI	3	n.a.	e3/e4
AD6	F	H	81	3	3	3	1.5	-/+	V-VI	3	12	e3/e3
AD7	F	W	94	3	3	3	0	-/+	V-VI	3	n.a.	e3/e3
AD8	F	W	85	3	3	3	1.5	-/+	V-VI	3	n.a.	e3/e3
AD9	M	W	66	3	3	3	1.5	-/+	V	3	19	n.a.
AD10	M	W	88	3	3	3	1.5	-/+	V-VI	1	16	e3/e2
AD11	F	W	86	3	3	2	1	-/+	V-VI	3	18	e3/e4
AD12	F	W	90	2	3	3	1	-/+	V	2	9	n.a.
AD13	F	W	100	2	3	3	0.5	-/+	V-VI	2	16	n.a.
AD14	M	W	88	2	3	2	1	-/+	V-VI	1	4	e3/e4
AD15	F	A	88	2	3	3	1.5	-/+	V	3	4	n.a.
MCI1	F	W	89	1	2	2	1	-/+	III-IV	0.5	24	e3/e3
MCI2	M	H	80	3	3	2	1	-/+	V	3	29	e3/e3
MCI3	M	W	93	2	0	2	0	-/+	0	3	19	e3/e2
MCI4	F	W	93	3	2	2	2	-/+	IV	3	11	e3/e3
MCI5	F	W	87	3	3	3	1.5	-/+	V-VI	3	13	e3/e3
MCI6	F	W	86	3	1	3	0	-/-	I-II	2	15	e3/e4
MCI7	F	W	76	3	3	3	2	-/+	V	1	26	e3/e4
MCI8	F	W	98	2	3	2	2	-/-	V	2	15	n.a.
MCI9	M	W	88	1	2	2	0	-/+	III	3	n.a.	n.a.
MCI10	F	B	94	2	1	2	0	-/-	I-II	0.5	29	e3/e3
CN1	M	W	95	1	1	1	0	-/-	I	0	30	e3/e3
CN2	F	W	99	1	2	1	0	-/-	III	0	29	e3/e3
CN3	M	H	81	3	1	2	0.25	-/-	I-II	0	23	e3/e4
CN4	F	W	93	3	2	3	0.5	-/+	III-IV	1	27	e3/e2
CN5	F	W	66	n.a.	n.a.	n.a.	n.a.	n.a.	n.a.	n.a.	n.a.	n.a.
CN6	M	W	84	n.a.	n.a.	n.a.	n.a.	n.a.	n.a.	n.a.	30	n.a.
CN7	F	B	73	n.a.	n.a.	n.a.	n.a.	n.a.	n.a.	n.a.	n.a.	n.a.
CN8	F	W	58	n.a.	n.a.	n.a.	n.a.	n.a.	n.a.	n.a.	30	n.a.
CN9	F	W	76	n.a.	n.a.	n.a.	n.a.	n.a.	n.a.	n.a.	n.a.	n.a.
CN10	F	W	75	n.a.	n.a.	n.a.	n.a.	n.a.	n.a.	n.a.	n.a.	n.a.
CN11	M	W	70	n.a.	n.a.	n.a.	n.a.	n.a.	n.a.	n.a.	30	n.a.
CN12	F	W	86	n.a.	n.a.	n.a.	n.a.	n.a.	n.a.	n.a.	n.a.	n.a.

Diagnosis	Sex	Race	Age at death	Thal A	Braak B	CERAD C	CAA Score	Co-morbid. [LB/ASVD]	Braak Stage	CDR Score	MMSE Score	APOE status
CN13	M	W	77	n.a.	n.a.	n.a.	n.a.	n.a.	n.a.	n.a.	30	n.a.
CN14	F	W	95	1	0	0	0.5	-/-	I	0	30	n.a.
CN15	M	W	69	0	0	1	0	-/+	0	1	28	n.a.
CN16	F	W	91	2	2	2	0	-/+	III	2	29	n.a.

AD, Alzheimer's disease dementia; MCI, mild cognitive impairment; CN, cognitively normal; F, female; M, male; A, Asian; B, Black; H, Hispanic; W, White; Thal A, Aβ plaque score modified from Thal; Braak B, NFT stage modified from Braak; CERAD C, Neuritic plaque score modified from CERAD; CERAD, Consortium to Establish a Registry for Alzheimer's Disease; CAA, Cerebral amyloid angiopathy; LB, Lewy bodies; ASVD. Atherosclerotic vascular disease; CDR, Clinical dementia rating; MMSE, Mini-Mental State Examination; n.a., not available; +: present; –: none; APOE, apolipoprotein E alleles.

Supplementary Table 2. List of antibodies for immunohistochemistry.

Primary antibody	Source Species	Dilution	Application	Commercial Source	Catalog Number
RBPMS pAb	Rabbit	1:300	IF	GeneTex	GTX118619
RBPMS mAb	Mouse	1:2000	IF	Invitrogen	MA5-26397
Parvalbumin pAb	Goat	1:200	IF	Novus Biologicals	NB100-1541
CCasp3 pAb	Rabbit	1:400	IF	Cell Signal	9661
VGLUT1 pAb	Guinea Pig	1:1000	IF	Chemicon	AB5905
Ser396 (pS396-tau) pAb	Rabbit	1:1200	IF, DAB	Anaspec	AS-54977
T22 (Oligo-tau) pAb	Rabbit	1:200	IF	Dr. Rakez Kayed Lab	-
PHF-1-tau mAb	Mouse	1:200	IF	Dr. Peter Davies Lab	-
scFvA13 (Oligo-Aβ) mAb	Mouse*	1:450	IF	Dr. Giovanni Meli Lab	-
12F4 ($A\beta_{42}$) mAb	Mouse	1:500	IF	Biolegend	805502
Phospho-MLKL mAb	Mouse	1:100	IF	R&D	MAB9187
CHMP2B mAb	Mouse	1:100	IF	R&D	MAB7509
CHMP2B pAb	Rabbit	1:200	IF	abcam	Ab33174
Secondary antibody					
Cy2 (anti-Goat, Mouse, Rabbit)	Donkey	1:200	IF	Jackson ImmunoResearch Laboratories	
Cy3 (anti-Goat, Mouse, Rabbit, Guinea Pig, Sheep)	Donkey	1:200	IF		
Cy5 (anti-Goat, Mouse, Rabbit, Guinea Pig, Sheep)	Donkey	1:200	IF		

Abbreviations: IF – immunofluorescence; DAB – peroxidase-based immunohistochemistry visualized with DAB (3, 3'-diaminobenzidine) substrate; Cyanine dyes – Cy2, Cy3, Cy5; pAb – polyclonal antibody; mAb – monoclonal antibody; p-tau – hyperphosphorylated tau; oligo-tau – oligomeric tau forms; PHF – paired-helical filament (pS396/pS404); scFv – single chain Fv fragment VGLUT1 – Vesicular glutamate transporter 1; *mouse recombinant antibody fragment.

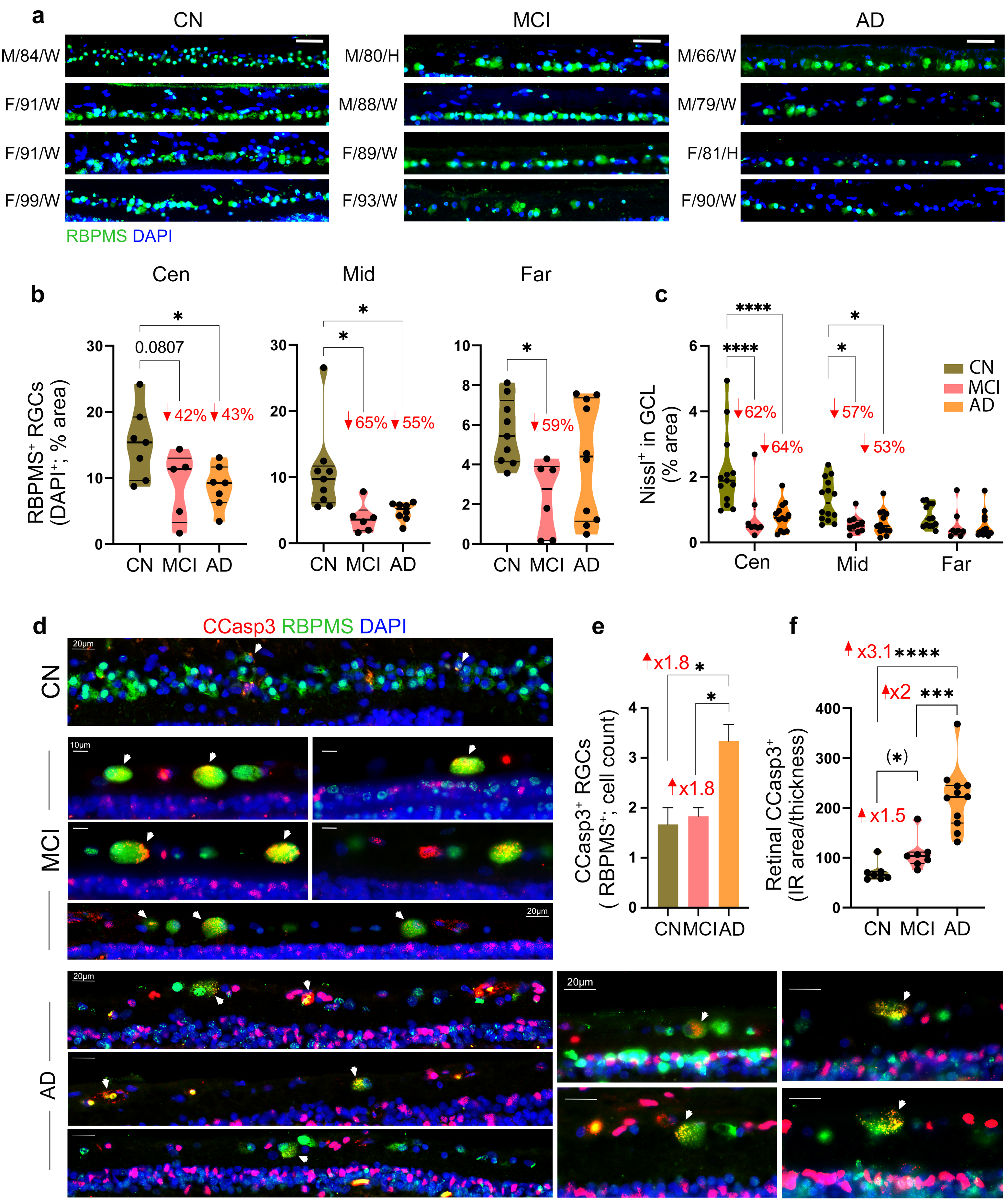

Supplementary Figure 1. Extended data on ganglion cell integrity in retinal tissues from MCI and AD patients.

Supplementary Figure 1. Extended data on ganglion cell integrity in retinal tissues from MCI and AD patients.

a Representative microscopic image of RGCs within the GCL, labeled with RBPMS (green) and nuclei DAPI (blue), in retinal cross-sections from patients with mild cognitive impairment (MCI due to AD, n=4) and Alzheimer's disease (AD) dementia (n=4), and cognitively normal (CN) individuals (n=4). Scale bar: 50µm. **b** Violin plots display quantitative immunohistochemistry analyses of $RBPMS^+DAPI^+$ RGCs percent area in Central (Cen), Mid-peripheral (Mid), and Far-peripheral (Far) subregions in the total ST region (n=25 subjects; n=9 CN, n=6 MCI, n=10 AD). **c** Quantitative analyses of $Nissl^+$ percent area in Central, Mid-, and Far-peripheral subregions (n= 33-37). **d** Representative microscopic images of the early apoptotic cell marker, cleaved caspase-3 ($CCasp3^+$, red) in $RBPMS^+$ cells (green) and nuclei DAPI (blue) in the GCL of CN, MCI, and AD donors. Colocalization of CCasp3 within $RBPMS^+$ RGCs (yellow) is indicated by white arrows. Scale bars: 10 and 20 µm. **e** Quantitative analysis of $CCasp3^+RBPMS^+$ RGC count in a subset of the same cohort. **f** Total retinal $CCasp3^+$ immunoreactive area analysis normalized to retinal thickness (n=25 subjects; n=7 CN, n=7 MCI, n=11 AD). Individual data points (circles) and median, lower and upper quartile are shown in violin plots. *P < 0.05, ***P < 0.001, ****P < 0.0001, by one-way ANOVA with Tukey's post-hoc multiple comparison test or unpaired Student t-test (in parenthesis). Percent decreases and fold changes are shown in red. F, Female; M, Male; Age (in years); Ethnicity: W, White and H, Hispanic; GCL, Ganglion cell layer; RGC, retinal ganglion cells.

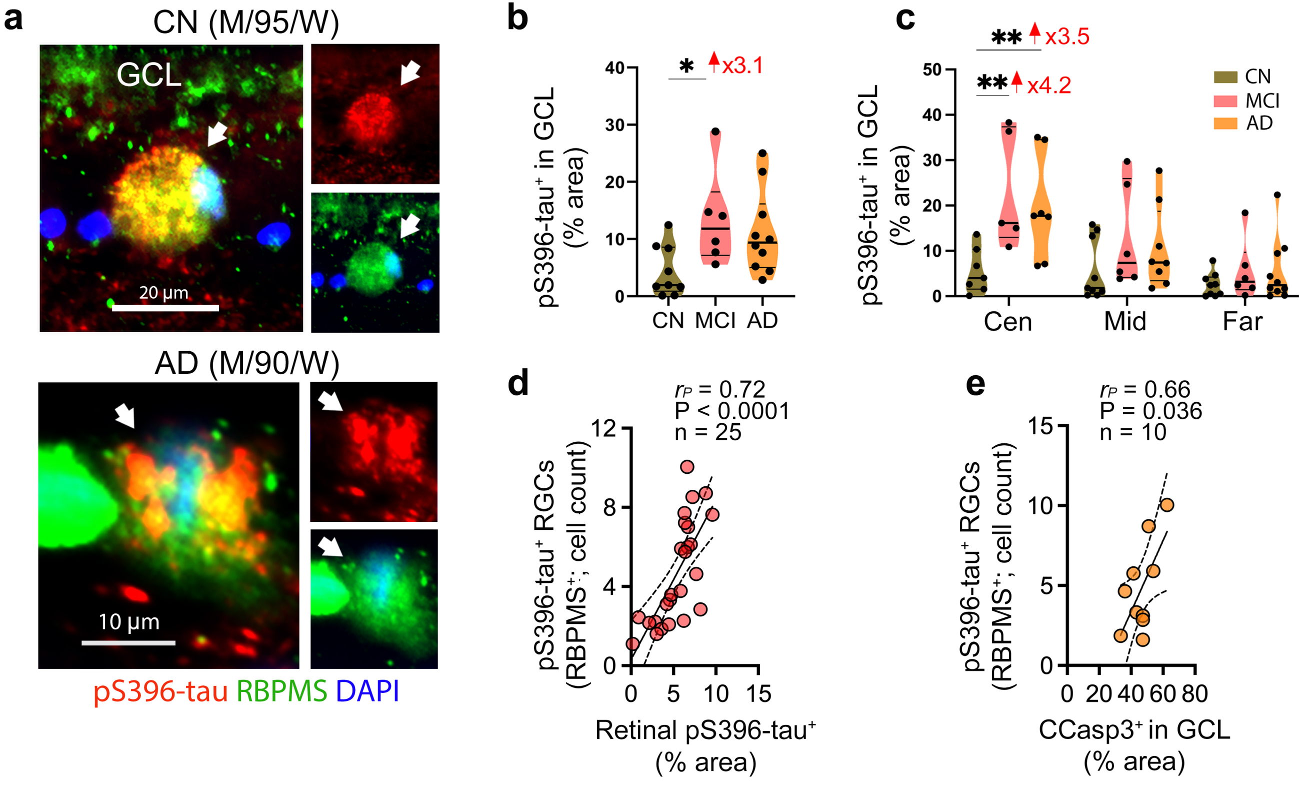

Supplementary Figure 2. Extended data on phosphorylated tau in RGCs of MCI and AD patients.

Supplementary Figure 2. Extended data on phosphorylated tau in RGCs of MCI and AD patients. **a** High-magnification microscopic images depicting pS396-tau accumulation (red) in swollen RBPMS^{+} RGCs (green) with hypertrophic soma (white arrows) in CN and AD retinas. Scale bar: 10µm. **b** Quantitative immunohistochemistry analysis of pS396-tau^{+} % area in the GCL (n=25 subjects; n=9 CN, n=6 MCI, n=10 AD). **c** Analyses of pS396-tau % area in the GCL in the Central, (Cen), Mid- peripheral (Mid) and Far-peripheral (Far) ST subregions (n=19-25). **d, e** Pearson's correlation (r_P) analyses between pS396-tau^{+} RGCs count and **d** retinal pS396-tau^{+} % area, **e** CCasp3^{+} % area in GCL. Individual data points (circles) and median, lower and upper quartile are shown in violin plots. *$P < 0.05$, **$P < 0.01$, by one-way or two-way ANOVA with Tukey's post-hoc multiple comparison test. Fold changes are shown in red. M, Male; Age (in years); Ethnicity: W, White. GCL, Ganglion cell layer; RGC, retinal ganglion cells.

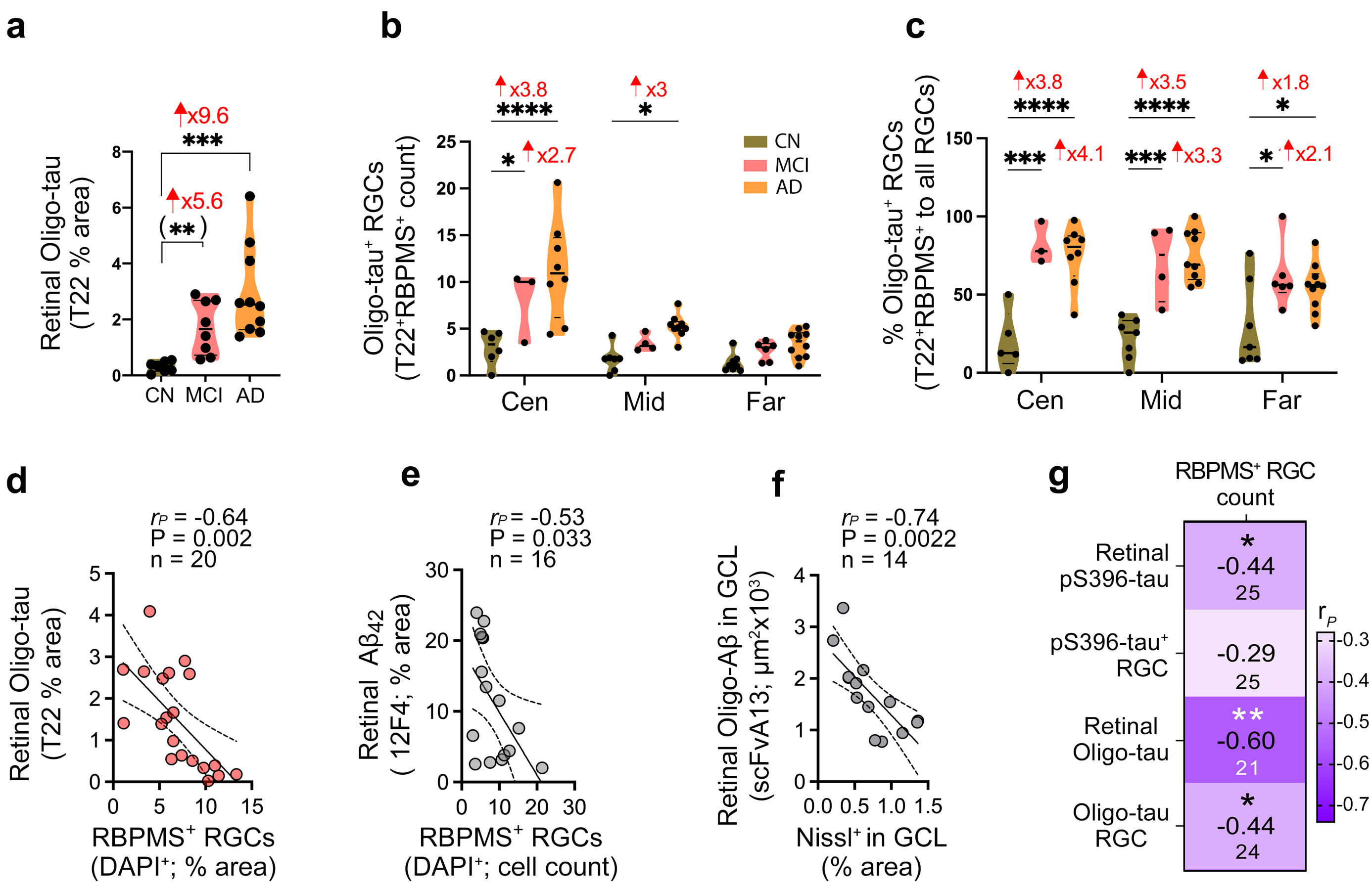

Supplementary Figure 3. Extended data on oligomeric tau isoforms within RGCs of MCI and AD patients.

Supplementary Figure 3. Extended data on oligomeric tau isoforms within RGCs of MCI and AD patients.

a Quantification of retinal T22$^+$ Oligo-tau percent area in MCI (n=8) and AD (n=10) patients vs. CN controls (n=10). **b** Cell count of Oligo-tau$^+$ RGCs in Cen, Mid-, and Far-peripheral retinal subregions (n= 13-27). **c** Percent of Oligo-tau$^+$ RBPMS$^+$ RGC count to total RBPMS$^+$ RGC count in Central (Cen), Mid- peripheral (Mid), and Far-peripheral (Far) retinal subregions (n=16-23). **d-f** Pearson's correlation (r_P) analyses between **d** retinal Oligo-tau percent area and RBPMS$^+$ RGCs percent area, **e** retinal $A\beta_{42}$ percent area and RBPMS$^+$ RGCs cell count, and **f** retinal Oligo-Aβ (scFvA13$^+$) area in GCL and Nissl$^+$ cells (in GCL) percent area. **g** Heatmap displays Pearson's correlations (r_P) of RBPMS$^+$ RGCs cell count with the following abnormal tau forms in the retina and within RGCs: retinal pS396-tau (% area), pS396-tau$^+$ RGCs (count), retinal T22$^+$ Oligo-tau (% area), T22$^+$ Oligo-tau$^+$ RGCs (% cell count). Large-font numbers indicate Pearson's *r* values and lower-font numbers indicate sample size (n). Individual data points (circles) and median, lower and upper quartile are shown in violin plots. *P < 0.05, ***P < 0.001, ****P < 0.0001 by one-way or two-way ANOVA with Tukey's post-hoc multiple comparison test or unpaired Student t-test (in parenthesis). Statistical significance shown in the heatmap is calculated by Pearson's correlation analyses. Fold changes are shown in red. GCL, Ganglion cell layer; RGC, retinal ganglion cells.

Supplementary Table 3. Correlations of pS396-tau or Oligo-tau RGCs with brain pathology and cognition in AD patients only.

**AD group**	Aβ (severity score)	NFT (severity score)	BRAAK (stage)	ABC (score)	CDR (score)	MMSE (score)
pS396-tau^{+} RGCs (count)	0.52*	0.46	0.32	0.49	0.29	-0.71*
Oligo-tau^{+} RGCs (count)	0.53*	0.56*	0.55*	0.72**	0.43	-0.70*

Spearman's rank correlations: P and *r*-values determine the statistical significance and strength of each pairwise association between pS396-tau^{+} or Oligo-tau^{+} RGCs with brain pathology and cognitive status in AD group only (comprises MCI due to AD and AD dementia patients). *P<0.05, **P<0.01. Aβ, amyloid beta-protein; CDR, Clinical Dementia Rating; NFTs, neurofibrillary tangles. ABC scores comprise of mean grades for: (A) Aβ plaque score modified from Thal, (B) NFT stage modified from Braak, and (C) neuritic plaque score modified from CERAD; MMSE, Mini-Mental State Examination.
